# A pontine-specific niche supports *de novo* gliomagenesis

**DOI:** 10.1016/j.celrep.2026.117796

**Published:** 2026-08-05

**Authors:** Adrija Pathak, Gregg B. Wells, Vytas A. Bankaitis, Zhigang Xie

**Affiliations:** 1 Department of Cell Biology & Genetics, Naresh K. Vashisht College of Medicine, Texas A&M University Health Science Center, College Station, TX 77843, USA; 2 Department of Chemistry, Texas A&M University, College Station, TX 77843, USA; 3 Lead contact

## Abstract

Pediatric high-grade gliomas (HGGs) exhibit genetic profiles accompanied by anatomical specificities. These features suggest that specific anatomical niches favor the onset and/or progression of these diseases. However, such niche identities remain elusive as existing genetically engineered mouse models fail to recapitulate these anatomical specificities. Herein, we investigate the anatomical specificities of histone H3.1 lysine 27-to-methionine (*H3.1K27M*) and activating *ACVR1* mutations for diffuse intrinsic pontine gliomas (DIPGs)—a major pons-specific pediatric HGG. We describe a murine model where expression of *H3.1K27M* and activating *Acvr1* and *Pik3ca* mutations (which co-occur with *H3.1K27M* in human DIPGs) induces rapid gliomagenesis specifically in the pons. We show that gliomagenesis occurs predominantly at the trigeminal root entry zone (TREZ) and requires hyaluronan receptor (HMMR) signaling. Our data demonstrate that anatomical specificities of human HGGs can be accurately recapitulated in mice and identify the TREZ as an anatomical niche that plays a key role in DIPG pathogenesis.

## In brief

Pathak et al. demonstrate that the anatomical specificities of pediatric high-grade gliomas can be reproduced in mice and that a pontine-specific structure plays a key role in the pathogenesis of a major subtype of this disease.

## Graphical abstract

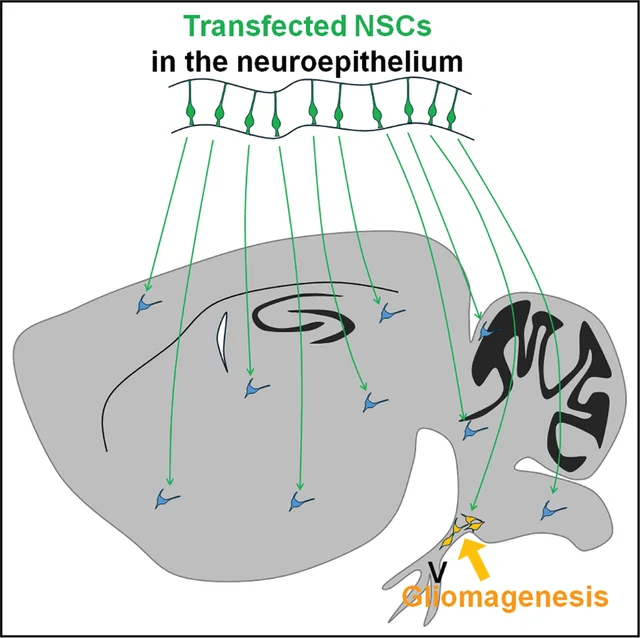

## INTRODUCTION

Brain cancer is the leading cause of cancer-related death in children and young adolescents.^1^ Among different types of pediatric brain cancers, high-grade gliomas (HGGs) are the most aggressive. Compared to adult HGGs, pediatric HGGs exhibit distinct genetic and anatomical features, such as the high incidence of the lysine 27-to-methionine (K27M) mutation in the histone H3.1 (*H3.1K27M*) or H3.3 genes (*H3.3K27M*) and the high proportion of gliomas in midline brain structures.^2–6^ The frequency of *H3.1K27M/H3.3K27M* is the highest in diffuse intrinsic pontine gliomas (DIPGs), a major subtype of pediatric HGGs.^7,8^ Of particular interest, an obligatory pair of *H3.1K27M* and activating *ACVR1* mutations occurs exclusively in DIPGs.^2–5^

Understanding how specific anatomical niches support the initiation and progression of pediatric DIPGs is central to devising rational strategies for treating these diseases. A major obstacle in such efforts is that genetically engineered mouse models (GEMMs) have thus far failed to recapitulate the anatomical specificities associated with genetic drivers of human disease.^9–15^ One possible reason for these failures is that the critical cell populations that give rise to *H3.1K27M/H3.3K27M*-induced gliomagenesis are not appropriately targeted in those models. In this scenario, activation of the driver mutations fails to satisfy dual conditions set by stemness and oligodendrocyte-lineage development programs. Both are key factors in DIPG pathogenesis.^9,11,14,16–19^

Herein, we assess the anatomical specificities of DIPG mutations in mice where the key *H3.1K27M*, activating *Acvr1*, and activating *Pik3ca* driver mutations are expressed at a particularly early developmental stage (embryonic day 11.5; E11.5). At this stage, the pons and the cerebral cortex are composed of a thin layer of neuroepithelium where early-stage neural stem cells (NSCs) are housed.^20,21^ We show that these mutations induce rapid gliomagenesis specifically in the trigeminal root entry zone (TREZ)—a ventrolateral pontine region where the trigeminal nerve enters the pons and intersects with other major axon tracts. We further demonstrate that signaling by HMMR, a hyaluronan receptor important for cell stemness, is critical for glioma cell proliferation in the TREZ.

## RESULTS

### Expression of H3.1K27M-AP driver mutations leads to *de novo* high-grade gliomagenesis in ventrolateral pons

To determine whether *H3.1K27M* and activated *Acvr1* drive pontine-specific gliomagenesis in mouse, these mutations were co-introduced into the brainstem. Tol2 transposon-based plasmids^22^ were designed for expressing H3.1-K27M and an activated ACVR1 (ACVR1-G356D).^4^ As activating mutations of phosphoinositide 3-kinase (PI3K) frequently co-occur with *H3.1K27M*/activated *ACVR1* in human DIPGs,^2–5^ a plasmid for expressing PIK3CA-E545K^23^ was also generated. These plasmids were introduced into the brainstem of mouse embryos via *in utero* electroporation together with a Tol2 transposon-based EGFP plasmid and a conventional plasmid for expressing the Tol2 transposase. The transposase catalyzed insertion of the various cassettes for expressing H3.1-K27M, ACVR1-G356D, PIK3CA-E545K, and EGFP (a combination we hereafter refer to as H3.1K27M-AP) into the genome of transfected cells. EGFP marked the transfected cells and their progeny.

**Figure 1.**
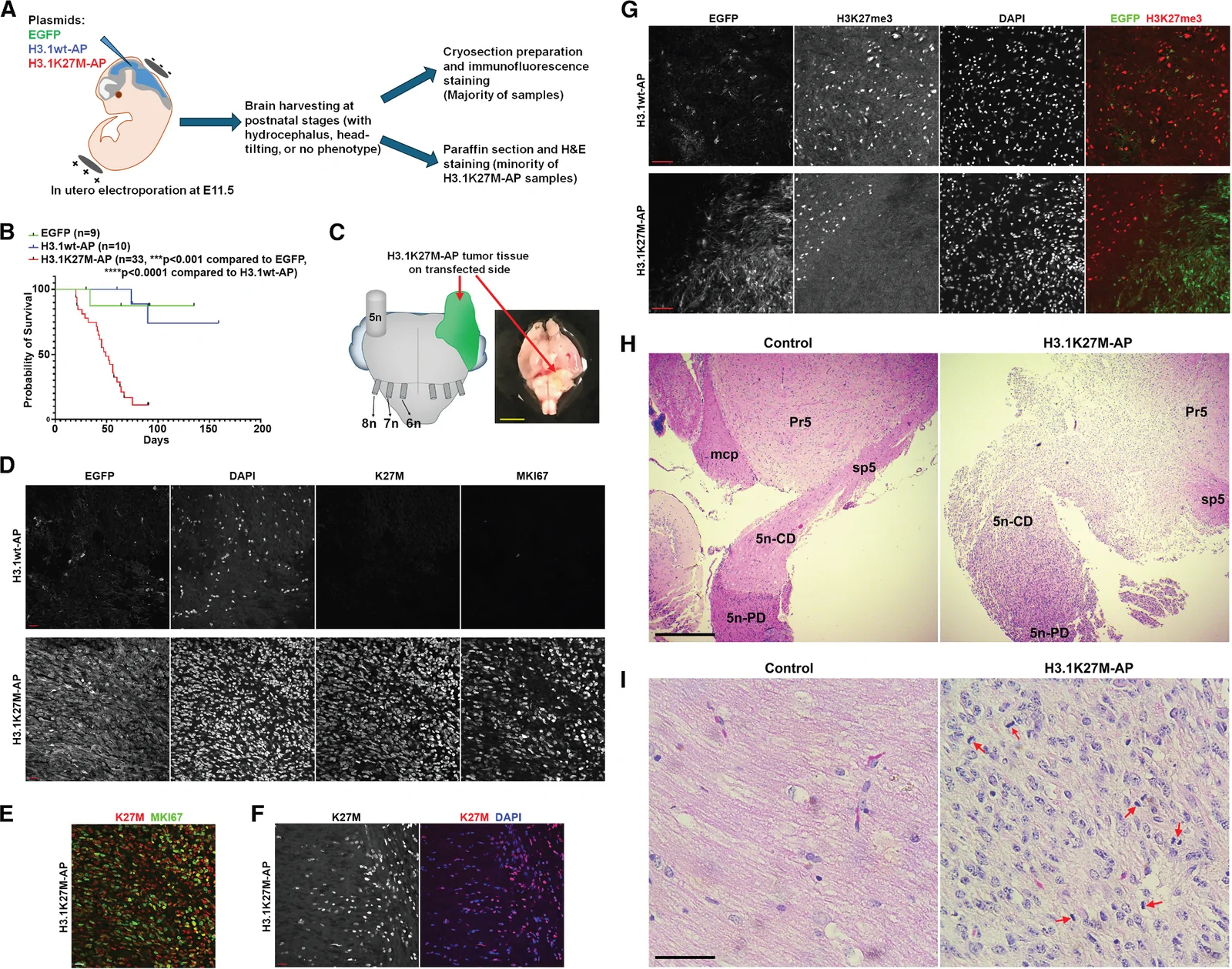
Expression of H3.1K27M-AP leads to *de novo* formation of high-grade tumors around the TREZ (A) Outline of the experimental procedures. (B) Kaplan-Meier survival analysis. ***p < 0.001, ****p < 0.0001, log rank test. (C) Location of the brain tumors induced by H3.1K27M-AP around the TREZ. A schematic representation of the tumor location is shown on the left side, and a representative brain sample (P52) with H3.1K27M-AP-induced tumor is shown on the right side. Scale bars: 5 mm. (D) Confocal images of the tumor bulk in the H3.1K27M-AP group and the center of a transfected area in the H3.1wt-AP group. Images are representative of 10 H3.1wt-AP mice at the age of P60–P159 and of 9 H3.1K27M-AP mice at the age of P40–P90. Scale bars: 10 μm. (E) The MKI67 panel and the K27M panel of the H3.1K27M-AP group in (D) are merged to show that nearly all proliferating cells (MKI67^+^ cells) are K27M^+^. Scale bars: 10 μm. (F) Confocal images at the edge of an H3.1K27M-AP tumor bulk (right). Tumor cells are identified by K27M immunoreactivity. Scale bars: 10 μm. (G) Confocal images of the edge of an H3.1K27M-AP tumor and the center of a transfected area in the H3.1wt-AP group. In the H3.1wt-AP group, EGFP^+^ cells exhibit H3K27me3 immunoreactivity similar to that of EGFP^−^ cells. In the H3.1K27M-AP group, H3K27me3 immunoreactivity is absent in tumor cells (EGFP^+^) but is detected in surrounding non-transfected cells (EGFP^−^; upper left corner). The images are representative of three H3.1wt-AP samples and four H3.1K27M-AP samples at the age of 1.5–3 months. Scale bars: 50 μm. (H and I) H&E images showing a tumor of the H3.1K27M-AP group. The tumor is located around the TREZ. The borders between distinct anatomical structures at this area can be clearly identified in the control sample but not in the H3.1K27M-AP sample due to the diffuse spread of the tumor cells (H). At higher magnifications, hypercellularity and high frequency of mitotic cells (arrows) are observed in the H3.1K27M-AP tumor tissue (I). Images are representative of six H3.1K27M-AP tumor samples at the age of 1.5–2 months and six age- and sex-matched non-transfected control samples. Similar anatomical areas (5n-CD) between the control and the H3.1K27M-AP group are shown. Scale bars: 500 μm in (H) and 50 μm in (I). Abbreviations are used as follows: 5n-CD, central domain of the trigeminal nerve; 5n-PD, peripheral domain of the trigeminal nerve; mcp, middle cerebellar peduncle; Pr5, principal sensory nucleus of the trigeminal nerve; sp5, spinal trigeminal tract. See also Figure S1 and Video S1.

Considering the importance of cell stemness in DIPG pathogenesis,^11,16,17^ these plasmids were introduced into the brainstem at the earliest stage where we could reliably perform *in utero* electroporation (E11.5). We reasoned that the mutations so introduced would be expressed in early-stage NSCs or progenitor cells and would therefore be more likely to induce or maintain high stemness in the transfected cells. Electroporated embryos were allowed to develop to term, and pontine gliomagenesis was assessed at various postnatal stages (Figure 1A). As a control (designated as H3.1wt-AP), the same procedure was performed with the exception that the *H3.1K27M* plasmid was replaced with a Tol2 transposon-based plasmid for expressing wild-type H3.1. In addition, some embryos were electroporated with a plasmid mixture for expressing EGFP alone.

Most mice born from electroporated embryos of the H3.1K27M-AP group displayed signs of DIPGs/brain tumors at the age of ~1.5–2 months. Phenotypes included hydrocephalus (18 out of 33) and head tilting (9 out of 33; one of these also displayed hydrocephalus) (Table S1; Video S1). These mice were promptly euthanized upon elaboration of these phenotypes. None of the mice in the control groups (EGFP or the H3.1wt-AP group) exhibited the head-tilting phenotype. However, a small number (1 out of 9 EGFP control and 2 out of 10 H3.1wt-AP control) developed hydrocephalus. These results suggest that the experimental procedure was a contributing factor to the hydrocephalus but a minor one. By contrast, the occurrence of hydrocephalus was significantly more frequent in the H3.1K27M-AP group compared to control groups (*p* value = 0.025; Fisher-Freeman-Halton exact test). Those results indicate that expression of H3.1K27M-AP mutations contributed to the hydrocephalus phenotype. Overall, the survival of the H3.1K27M-AP group was markedly shorter than that of control groups (Figure 1B).

Half of the mice in the H3.1K27M-AP group (17/33) had already developed large, visible tumor bulk at the time of harvest. These tumor bulks were located in the ventrolateral pontine area on and surrounding the trigeminal nerve (Figure 1C). In the remaining H3.1K27M-AP mice, clusters of transfected cells with high rates of proliferation were observed in the same areas. Immunostaining analyses revealed that the tumor bulks in the H3.1K27M-AP group were characterized by high cellularity, expression of the K27M mutant histone H3 protein (>99% of the proliferating cells were K27M^+^ in all tumor samples), and high rates of cell proliferation (51.1% ± 13.8% of K27M^+^ cells were MKI67^+^; mean ± SD, *n* = 5 mice) (Figures 1D and 1E). The MKI67^+^K27M^+^ cells were present throughout the lateral pons and were most concentrated in the TREZ area and the pons surface (Figure S1A). MKI67^+^K27M^+^ cells also invaded the medial ventral pons via the middle cerebellar peduncle and the surface of the ventral pons (Figure S1A). Thus, tumor cell distribution within the brainstem was primarily concentrated in the TREZ region with some tumor cells having migrated out via axon tracts or exophytic spread.

Examination of tumor bulk edges indicated that K27M^+^ cells diffused into the nearby non-transfected tissue with no clear border between the tumor bulk and surrounding normal tissue (Figure 1F). Moreover, immunostaining analyses demonstrated that the tumor tissue exhibited clear loss of the H3K27me3 epigenetic mark (Figure 1G). In control mice (EGFP alone or H3.1wt-AP), no dense clusters of proliferating cells were detected around the transfected area or in any other part of the brainstem (Figures S1B and S1C). However, scattered proliferating cells were observed in some areas of the brainstem.

Interestingly, a small subset of mice in the H3.1K27M-AP group harbored proliferating K27M^+^ cells that did not show EGFP expression (Figures S1D and S1E). Although why EGFP expression was suppressed in these cells remains unclear, the data indicated that EGFP expression alone was insufficient for confident identification of all transfected cells and their progeny. Thus, expression of K27M was preferentially used in the following analyses when compatible antibodies were available for co-immunostaining. While this method could not be used for control groups where no K27M was expressed, no increased cell proliferation was observed in any brainstem region in the H3.1wt-AP group compared to the group that expressed EGFP alone (Figures S1B and S1C). Thus, the tumor cell proliferation observed in the H3.1K27M-AP group was *H3.1K27M* dependent.

### H3.1K27M-AP tumors exhibit properties characteristic of human DIPGs

Examination of H3.1K27M-AP tumor tissue histopathology in hematoxylin and eosin (H&E)-stained paraffin sections revealed features typical of HGGs, such as dense hypercellularity, poorly differentiated tumor cells, marked nuclear pleomorphism, and a high density of mitotic figures (Figures 1H and 1I). The activation of ACVR1 and PI3K pathways was evidenced by increased immunoreactivity of phospho-SMAD1/5/9 and increased immunoreactivity of Ser473-phosphorylated AKT in H3.1K27M-AP tumor tissue relative to non-transfected tissues and to tissues from the EGFP-transfected control group (Figures S2A and S2B, respectively). Expression of Ser28-phosphorylated histone H3 (a mitosis marker) confirmed the high frequency of mitotic cells in the tumor tissues (Figure S2C). Increased expression of an angiogenesis marker ANGPT1^24^ was also evident (Figure S2D). These data suggest that angiogenesis was induced to some degree to support tumor growth, even though glomeruloid vascular proliferation typical of glioblastoma was not observed in the H3.1K27M-AP tumor tissues.

**Figure 2.**
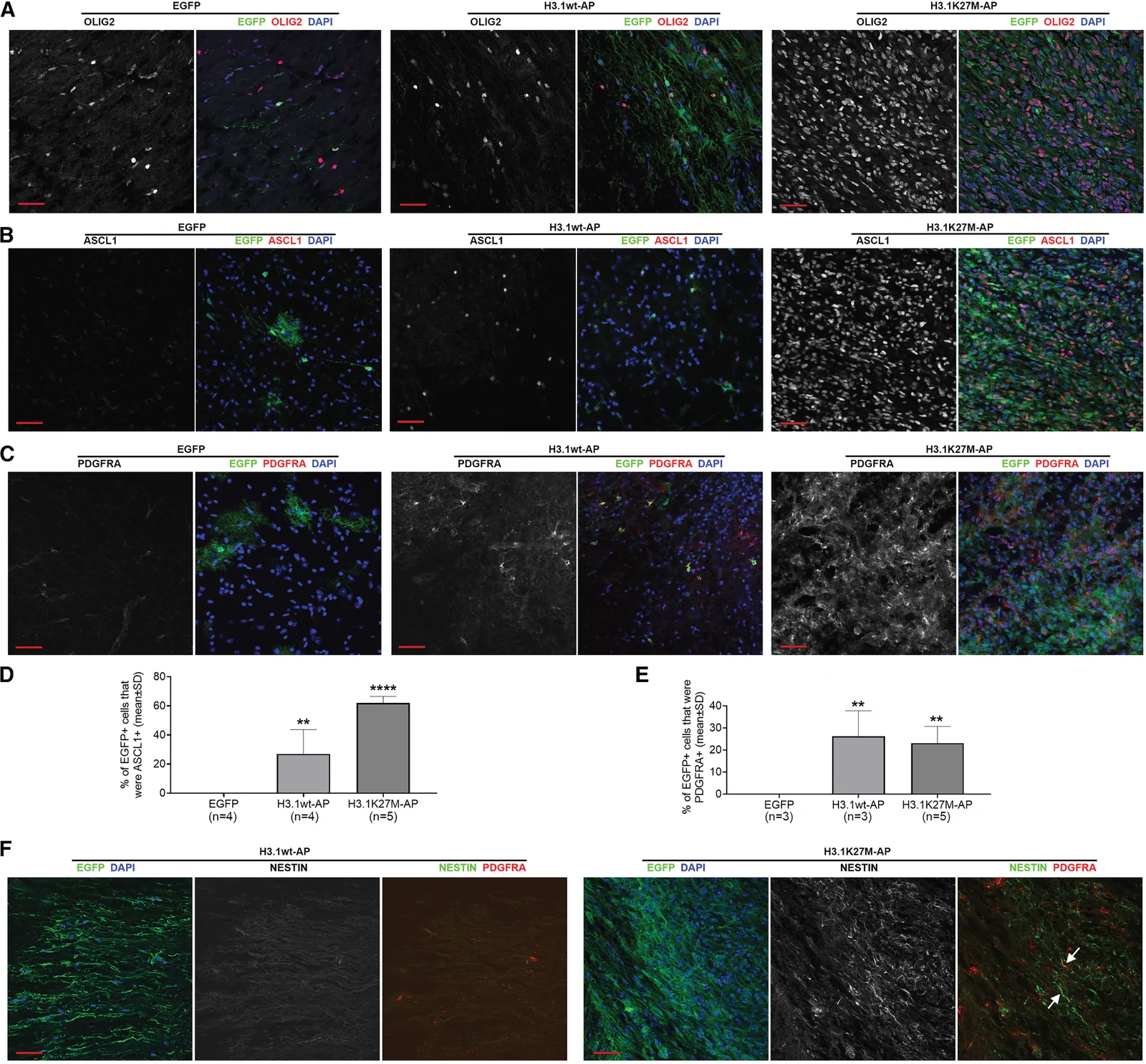
Tumor cells induced by H3.1K27M-AP express markers of oligodendrocyte-lineage cells and NSCs (A) Most transfected cells in the H3.1K27M-AP group and some cells in control groups express OLIG2. Scale bars: 20 μm. (B and C) Overexpression of ASCL1 and PDGFRA in the H3.1K27M-AP group and the H3.1wt-AP group but not in the EGFP control group. Scale bars: 20 μm. (D and E) Quantifications of ASCL1 and PDGFRA expression in transfected cells. The *n* values indicate the number of mice quantified. **p < 0.01, ****p < 0.0001, one-way ANOVA. The relatively large standard deviation in (D) might result from variations in the ability of the H3.1wt-AP plasmid combination to promote ASCL1 expression among different samples. (F) Overexpression of NESTIN in the H3.1K27M-AP group but not in the H3.1wt-AP group. Some cells overexpress both NESTIN and PDGFRA (indicated by arrows). The weak fluorescence in the H3.1wt-AP NESTIN panel is non-specific background staining. Scale bars: 20 μm. See also Figure S2. Confocal images of the H3.1K27M-AP samples and control samples (EGFP and H3.1wt-AP; from ventrolateral pontine areas corresponding to the site of tumorigenesis in H3.1K27M-AP samples) representative of 3 or more samples at the age of 1–3 months for each group are shown.

Finally, H3.1K27M-AP tumor gene expression data exhibited signatures that recapitulate those in human DIPGs. These tumor cells expressed markers of oligodendrocyte-lineage cells (e.g., OLIG2, ASCL1, and PDGFRA; Figures 2A–2E). Nearly all proliferating tumor cells expressed OLIG2 (98.47% ± 0.97%; mean ± SD, *n* = 6 mice) (Figure S2E). Expression of an NSC marker NESTIN was also evident in tumor tissues (Figure 2F). Notably, glial cell-lineage markers (ASCL1, PDGFRA, and GFAP) were also expressed or overexpressed in the H3.1wt-AP group (Figures 2A–2E; Figure S2F). Only the NSC marker NESTIN was specifically expressed in the H3.1K27M-AP group but not in the H3.1wt-AP group (Figure 2F). These data are consistent with previous reports that activated ACVR1 alone arrests glial cell differentiation^14^ and that the H3K27M mutation enhances NSC stemness.^11^ Finally, the tumor mass was marked by extensive infiltration of immune cells as reported by the prominent CD45 immunoreactivity (Figure S2G).

Taken together, the collective results indicate that tumors in the H3.1K27M-AP group exhibit characteristics of HGGs consistent with human diffuse midline gliomas (DMGs), H3K27-altered (current umbrella term encompassing DIPGs that harbor H3K27M mutations). These include dense hypercellularity, poorly differentiated tumor cells, marked nuclear pleomorphism, high rates of proliferation, K27M expression coupled with loss of H3K27me3, diffuse distribution of tumor cells, and expression of markers of NSCs, glial lineage cells, and immune cells.

### H3.1K27M-AP-induced gliomas proliferate on axon tracts in the TREZ

**Figure 3.**
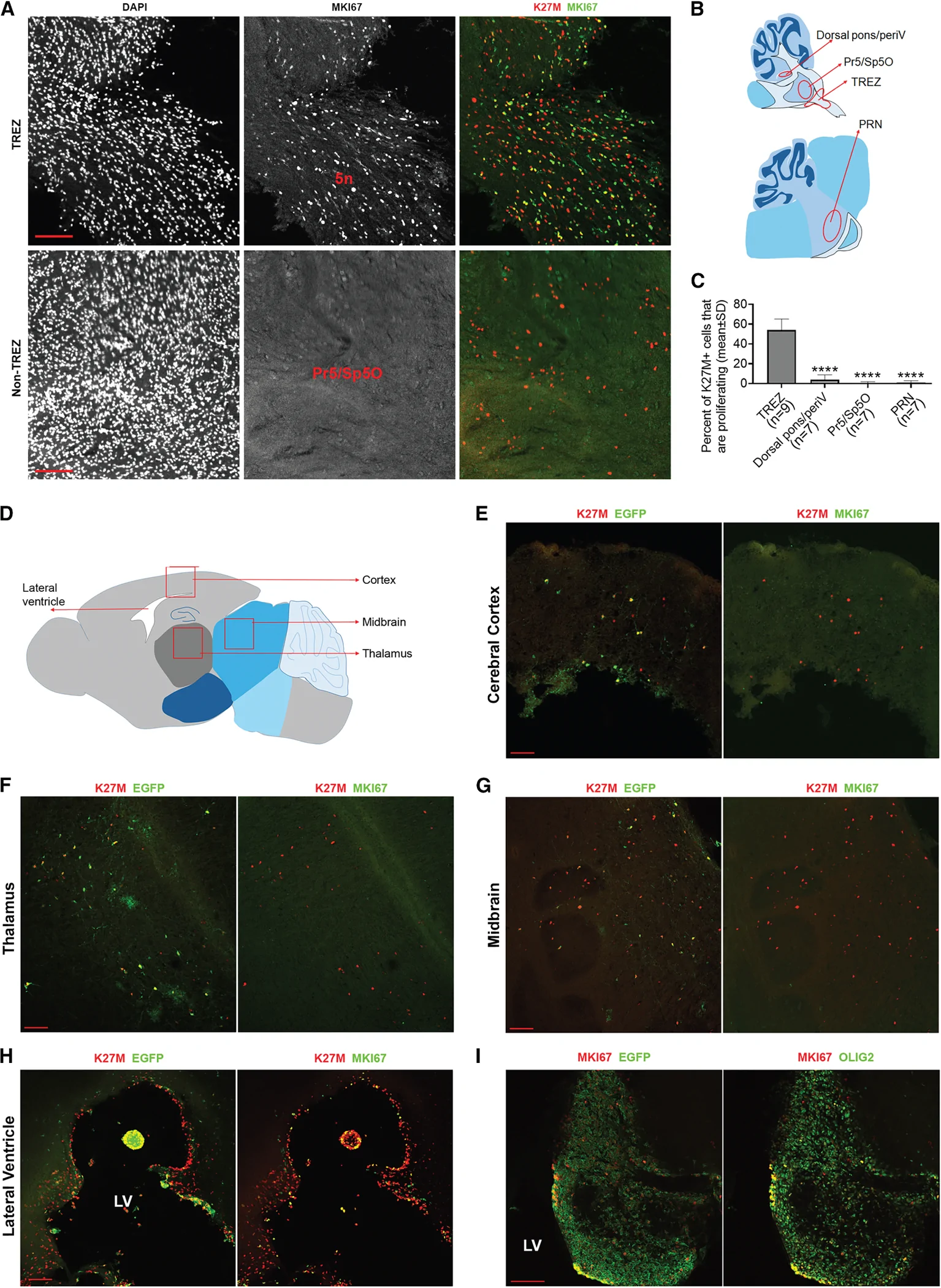
The TREZ is the predominant site of glioma cell proliferation driven by H3.1K27M-AP (A–C) Comparison of the proliferation rate of glioma cells in the TREZ and non-TREZ areas of the brainstem. Confocal images of a P21 mouse expressing H3.1K27M-AP are shown in (A), a schematic illustrating the areas used for quantification is shown in (B), and quantifications are shown in (C). Transfected cells were identified by K27M immunoreactivity. Scale bars: 100 μm. ****p < 0.0001, one-way ANOVA. (D–I) Analysis of cell proliferation in experiments of forebrain/midbrain electroporation. Typical transfected regions are illustrated in (D). Images are representative of 13 mice (P21–P135) for the cerebral cortex group (E), six mice (P21–P47) for the thalamus group (F), five mice (P25–P69) for the midbrain group (G), and two mice (P73 and P130; out of the 13 forebrain-transfected mice) for the periventricular/ventricular gliomagenesis group (H). Scale bars: 100 μm. (I) Expression of OLIG2 in periventricular/ventricular gliomas induced by H3.1K27M-AP. Confocal images of tumor cells at the periventricular/ventricular area from a P73 mouse are shown. Images are representative of two mice in which forebrain expression of H3.1K27M-AP-induced periventricular/ventricular tumor growth. Scale bars: 100 μm. Abbreviations are used as follows: 5n, trigeminal nerve; LV, lateral ventricle; PeriV, periventricular area; Pr5, principal sensory trigeminal nucleus; PRN, pontine reticular nucleus; Sp5O, spinal trigeminal nucleus, oral part; TREZ, trigeminal root entry zone. See also Figure S3.

Most of the H3.1K27M-AP mice that displayed hydrocephalus were euthanized before large infiltrative tumors formed. The euthanized mice were analyzed for the anatomical specificity of glioma cell proliferation induced by H3.1K27M-AP because the dispersion of tumor cells was limited. We also assessed the anatomical specificities of cell proliferation in mice transfected with H3.1K27M-AP at relatively low efficiencies (by adjusting the parameters of the *in utero* electroporation procedure) that did not display hydrocephalus. The anatomical locations were identified based on the overall mediolateral and dorsoventral position of the sections and the spatial arrangement of the DAPI-labeled cell nuclei (Figures S2H and S2I). Strikingly, the TREZ defined the predominant site of cell proliferation in all of these mice (ages P20–P91; Figures 3A–3C). Compared to other regions, the TREZ harbored the largest concentration of proliferating cells and the highest fraction of proliferating K27M^+^ cells. Few proliferating cells were observed in regions not directly connected to the TREZ via major axon tracts (Figures 3A–3C). While K27M^+^ cells also proliferated on axon tracts away from (but directly connected to) the TREZ, the total numbers of transfected cells in those areas were far fewer than those at the TREZ (Figures S2J and S2K). We speculate that those cells were migrating along axon tracts from the TREZ or were rapidly migrating to the TREZ via axon tracts to continue persistent proliferation. Both possibilities are consistent with the TREZ representing the predominant site of cell proliferation.

### H3.1K27M-AP fails to induce gliomas in other brain regions

As the TREZ is a pontine structure, glioma cell proliferation induced by H3.1K27M-AP in this anatomical space offers a rationale for the specific detection of the H3.1K27M/activating ACVR1 mutation pair in human DIPGs. To further interrogate this idea, H3.1K27M-AP mutations were introduced into more rostral parts of the brain at the same embryonic stage (i.e., E11.5). H3.1K27M-AP failed to induce postnatal cell proliferation within the parenchyma of the cerebral cortex, in the thalamus, or in the midbrain (Figures 3D–3G). Interestingly, in a subset of mice (2 out of 13 animals with moderate to high transfection efficiencies at the forebrain), periventricular/ventricular tumorigenesis was induced (Figure 3H) with most of the proliferating tumor cells expressing OLIG2 (Figure 3I). In control mice where H3.1K27M was replaced with H3.1wt, no such periventricular/ventricular glioma-like cell proliferation was observed (Table S1). The induction of periventricular/ventricular gliomagenesis by H3.1K27M-AP remains consistent with the general concept that the H3.1K27M and activating ACVR1 mutation pair is specific to DIPGs, as H3K27M-positive periventricular/ventricular gliomas are reported to occasionally occur in human patients.^25–27^

### TREZ-specific gliomagenesis does not result from selective transfection of TREZ cells

**Figure 4.**
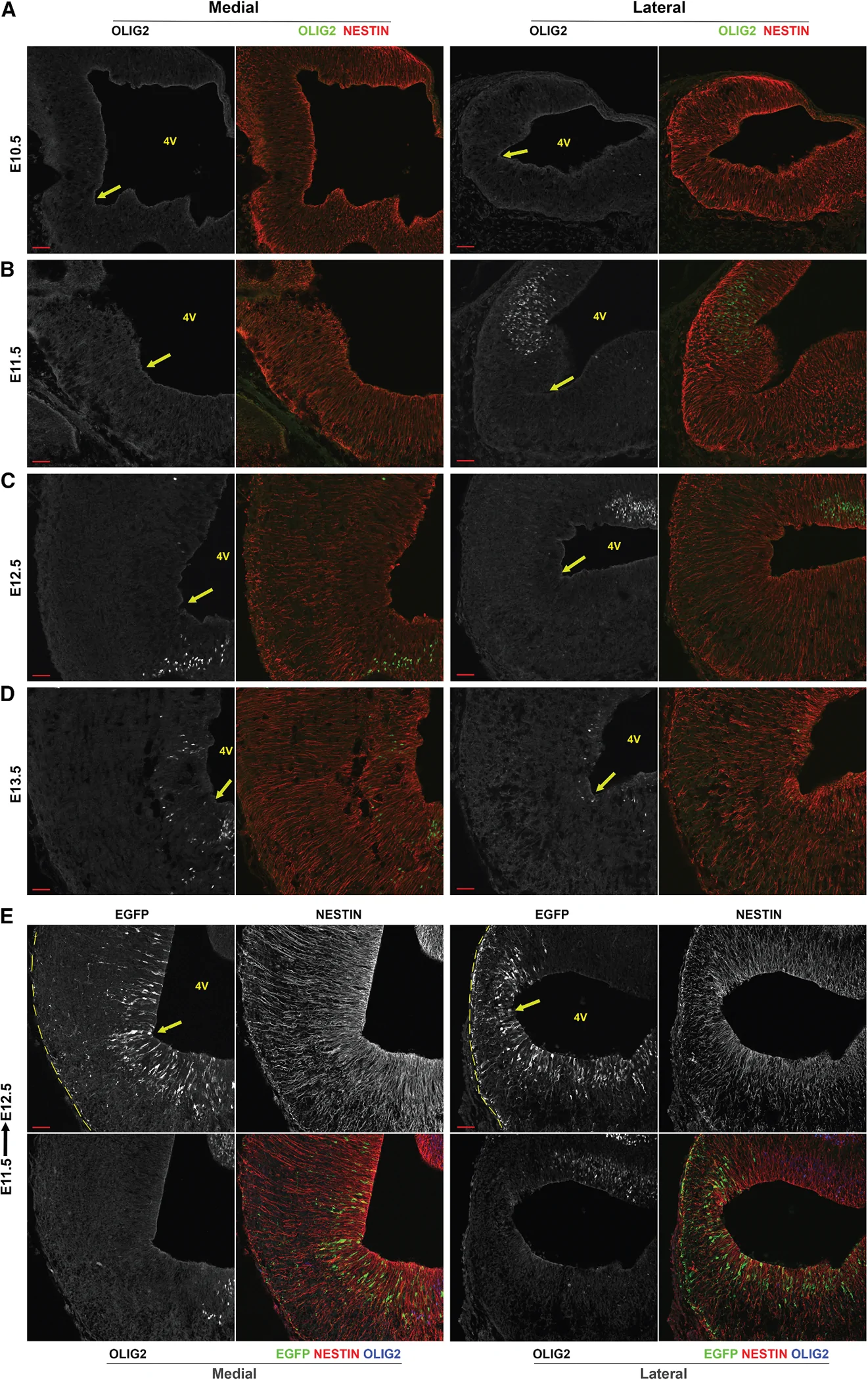
Expression of OLIG2 in the embryonic mouse pons Sagittal sections of the embryonic mouse hindbrain are shown. Arrows indicate the pontine flexure. NESTIN labels NSCs. (A–D) Immunostaining analysis of OLIG2. Scale bars 50 μm. 4 V: the fourth ventricle. (E) *In utero* electroporation at E11.5 leads to transfection of NSCs that do not express OLIG2. The pial surface is outlined by yellow dashed lines. Most of the EGFP fluorescence at the pial surface is from the tip of the basal process of the NSCs. Images are representative of four electroporated embryos. 4 V: the fourth ventricle. Scale bars: 50 μm.

Two different mechanisms might account for the TREZ-specific gliomagenesis induced by H3.1K27M-AP. One possibility is that H3.1K27M-AP expression is insufficient for gliomagenesis, but exposure to the TREZ enhances the gliomagenesis potential of H3.1K27M-AP stem or progenitor cells. Alternatively, H3.1K27M-AP expression may be sufficient to drive gliomagenesis in different anatomical locations, but our experimental procedure may lead to preferential transfection of OLIG2^+^ cells at the TREZ or those that migrate to the TREZ. To investigate this latter possibility, we first assessed OLIG2 expression in the embryonic mouse pons around the stage of our electroporation. OLIG2 immunoreactivity was absent in the brainstem at E10.5 (Figure 4A) but was detectable in regions more rostral and caudal to the pontine flexure at E11.5 and E12.5 (Figures 4B and 4C). OLIG2 immunoreactivity started to appear at the pontine flexure at E13.5 where rhombomeres give rise to a major part of the pons^28^ (Figure 4D). To determine the identities of the transfected cell populations, a transposon-based EGFP plasmid was co-electroporated with a conventional plasmid for expressing the Tol2 transposase into the embryonic mouse pons at E11.5. The embryos were subsequently harvested 24 h after electroporation. EGFP^+^ cells exhibited a broad distribution that covered the pontine flexure and extended into the rostral and caudal directions in both medial and lateral brainstem (Figure 4E). Most of these cells were radially aligned and bipolar in shape with the soma located in the ventricular zone (Figure 4E). The shapes and locations of these cells were consistent with those of NSCs (neuroepithelial and radial glial cells). Moreover, nearly all these cells scored as OLIG2^−^ (99.7% ± 0.7%; mean ± SD; *n* = 4 embryos) (Figure 4E). Thus, the experimental procedure led to the primary, if not exclusive, transfection of NSCs that did not express OLIG2 over an expansive area of the brainstem.

To further examine the TREZ specificity of gliomagenesis, the H3.1K27M-AP plasmid mixture was electroporated into the embryonic mouse brainstem at E11.5. The distributions and proliferation of transfected cells were then scored at several different developmental time points. At E15.5 and P4, transfected cells (K27M^+^ cells) were distributed throughout the pons with no significant difference in the proliferation rate between the transfected cells and neighboring non-transfected cells in any of the anatomical locations (Figures S3A–S3D). While the proliferation rates were similar between transfected cells and non-transfected cells in non-TREZ areas at P11, an increased proliferation rate was observed for transfected cells vs. non-transfected cells in the TREZ (Figures S3E and S3F). Those data reveal that H3.1K27M-AP *in utero* electroporation produces transfected cells throughout the pons without elevating proliferation in any brainstem areas (compared to non-transfected cells in the same areas). It is only at later time points (P4–P11) that increased proliferation was noted in transfected cells, and this effect was restricted to cells populating the TREZ.

### H3.3K27M-TPC-induced glioma-like cell proliferation in the brainstem outside of the TREZ

To further interrogate whether TREZ-specific gliomagenesis induced by H3.1K27M-AP reported an authentic biological specificity, *de novo* gliomagenesis was induced using genetic combinations that present different anatomical specificities. To that end, we chose a combination of *H3.3K27M* and its common DIPG co-occurring genetic elements that lead to gliomagenesis both in the pons and in other midline structures in human patients.^2–5^ If the anatomical specificity we observe for H3.1K27M-AP-induced gliomagenesis reflects a functional contribution of the TREZ, then expression of H3.3K27M and its DIPG co-occurring genetic elements will fail to recapitulate TREZ specificity. By contrast, reproduction of the TREZ specificity by expression of H3.3K27M and its DIPG co-occurring genetic elements would support a technical bias in the experimental protocol.

**Figure 5.**
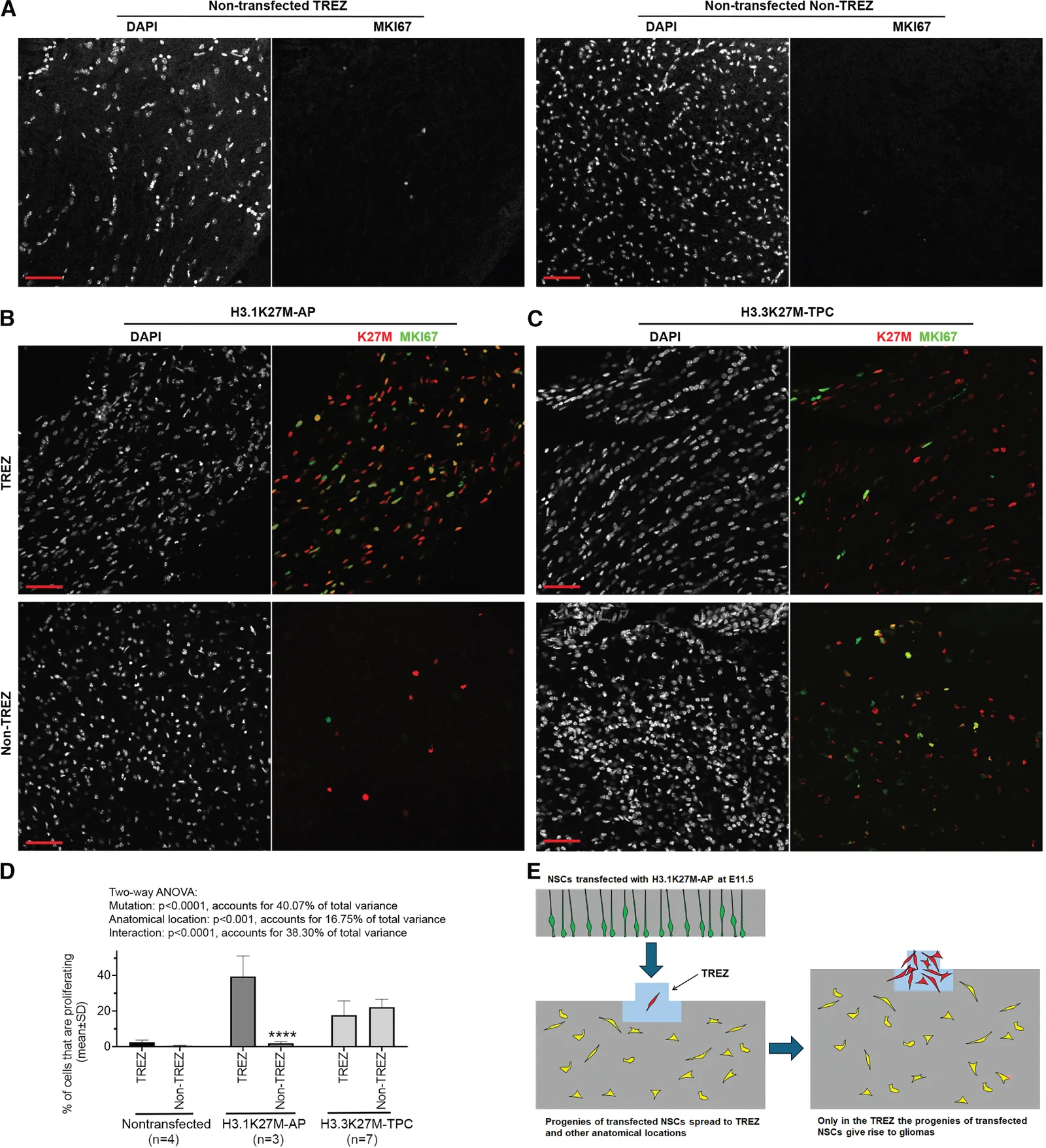
TREZ-specific glioma cell proliferation is dependent on genetic mutations Mouse embryos were electroporated at E11.5, and proliferation of transfected cells was analyzed at P20. Same areas from non-transfected P20 mice were also assessed. (A–C) Representative confocal images. The central domain of the trigeminal nerve (5n-CD) and the principal sensory nucleus of the trigeminal nerve (Pr5) are shown to represent the TREZ and the non-TREZ areas, respectively. Scale bars: 20 μm. (D) Quantification by two-way ANOVA analysis reveals that there is a significant interaction between genetic mutation (non-transfected, H3.1K27M-AP, or H3.3K27M-TPC) and anatomical location (TREZ vs. non-TREZ). ****p < 0.0001, non-TREZ compared to TREZ in the H3.1K27M-AP group. (E) A schematic model illustrating how H3.1K27M-AP induces *de novo* gliomagenesis in the TREZ. See also Figures S3, S4, and S5.

The most frequent genetic alterations co-occurring with *H3.3K27M* are *TP53* mutations and overexpression of the *PDGFRA* and *CDK/CCND* genes.^2–5^ We therefore designed Tol2 transposon-based plasmids for expressing *H3.3K27M*, *Trp53-R172H*,^29^ wild-type *Pdgfra*, and wild-type *Ccnd2* (hereafter referred to as H3.3K27M-TPC). These plasmids were electroporated into the brainstem of mouse embryos at E11.5 together with a Tol2 transposon-based EGFP plasmid and a conventional plasmid for expressing the transposase Tol2. Postnatal gliomagenesis in mice born from the electroporated embryos was subsequently analyzed at P20. We also conducted a parallel experiment with the H3.1K27M-AP plasmid mixture. The readout was a comparison of transfected cell proliferation within vs. outside the TREZ for the H3.1K27M-AP and H3.3K27M-TPC groups and for non-transfected cells. Whereas non-transfected cells exhibited low rates of proliferation within and outside of the TREZ, the H3.1K27M-AP group experienced a marked increase in cell proliferation specifically within the TREZ (Figures 5A–5D). By contrast, H3.3K27M-TPC substantially increased cell proliferation in areas within and outside of the TREZ (Figures 5A–5D).

The effect of H3.3K27M-TPC expression on gliomagenesis was further analyzed at later postnatal stages (~1.5–6 months). At these later stages, no clusters of transfected proliferating cells were apparent in the brainstem of mice that experienced moderate transfection efficiencies (Figures S4A and S4B). By contrast, mice with high transfection efficiencies presented glioma-like cell proliferation in the brainstem (Figures S4C and S4D). No appreciable cell proliferation was induced in the brainstem of control mice with high transfection efficiencies where H3.3-K27M was replaced with wild-type H3.3 (Figure S4C). These data demonstrate an *H3.3K27M*-dependence of glioma-like cell proliferation. Immunostaining analyses revealed two types of H3.3K27M-TPC-transfected proliferating cell clusters: those in which only a subset of proliferating cells expressed OLIG2 and those in which nearly all proliferating cells expressed OLIG2 (Figures S4E and S4F). Intriguingly, glioma-like cell proliferation was observed in various brainstem locations outside but not within the TREZ. This was the case even though high densities of K27M^+^ cells populated the TREZ (Figures S5A–S5C).

As an additional test of TREZ-specific H3.1K27M-AP-induced gliomagenesis, the wild-type PDGFRA expression plasmid in the H3.3K27M-TPC combination was replaced with one driving activated PDGFRA-D842V expression.^30^ This modified plasmid mixture (hereafter referred to as the D842V group) was electroporated into the embryonic mouse brainstem at E11.5. Consistent with previous reports of potent tumorigenic activity of PDGFRA-D842V,^9,10^ mice in the D842V group developed large tumor bulk in the brainstem at 3 weeks to 1 month. Examination of cell proliferation in the brainstem of these mice revealed that high rates of cell proliferation occurred both within and outside of the TREZ (Figures S5D–S5F). Two-way ANOVA analyses of cell proliferation among the H3.1K27M-AP group, the H3.3K27M-TPC group (at the age of ~1.5–6 months), and the D842V group confirmed a strong interaction between genetic background and anatomical location—that is, the effect of anatomical location on cell proliferation is strongly dependent on the genetic context, and vice versa (Figure S5F). In aggregate, these data demonstrate a physiological basis for the TREZ specificity of H3.1K27M-AP-induced pontine gliomagenesis (Figure 5E).

### HMMR signaling in H3.1K27M-AP-induced pontine gliomagenesis

The TREZ is characterized by the presence of large axon tracts. Given the cardinal signatures of cell stemness and the oligodendrocyte developmental program in DIPG pathogenesis,^9,11,14,16–19^ our data indicate that H3.1K27M-AP potentiates stemness of oligodendrocyte-lineage stem/progenitor cells. Such enhancement is in turn amplified in the uniquely supportive TREZ large axon tract niche. This concept predicts that interfering with cell stemness, and/or the interaction of oligodendrocyte-lineage cells with axons, will inhibit H3.1K27M-AP-induced gliomagenesis.

While the interactions between axons and oligodendrocyte-lineage cells might be mediated by both synaptic molecules and extracellular matrix (ECM) adhesion molecules,^31–34^ the large axon tracts of the TREZ generally form synapses with neurons that are located outside of the TREZ (e.g., the principal sensory nucleus of the trigeminal nerve). Thus, we sought to identify ECM signaling pathways as potential targets for interfering with H3.1K27M-AP-induced TREZ-specific gliomagenesis. Of all the ECM molecules and their receptors that have been implicated in the interactions between axons and oligodendrocyte-lineage cells,^31^ we focused on *Hmmr*. This gene encodes a receptor for the ECM hyaluronan and exhibits the unique properties of being a highly developmentally regulated gene with an established role in the stemness of NSCs and other stem cells.^35–40^ *Hmmr* transcript abundance in the central nervous system (CNS) is >10-fold higher at E11.5 than at E18 (based on the reads per kilobase million value for NCBI gene ID: 15366). Genes exhibiting this pattern of enrichment (e.g., the microcephaly genes *Aspm* [NCBI gene ID: 12316] and *Kif11* [NCBI gene ID: 16551]) often play important and specific roles in NSC stemness. We therefore considered the possibility that *Hmmr* regulates gliomagenesis at two levels: (1) by mediating the interaction between axons and oligodendrocyte-lineage cells and (2) by promoting NSC stemness.

### HMMR contributes to H3.1K27M-AP-induced pontine gliomagenesis

**Figure 6.**
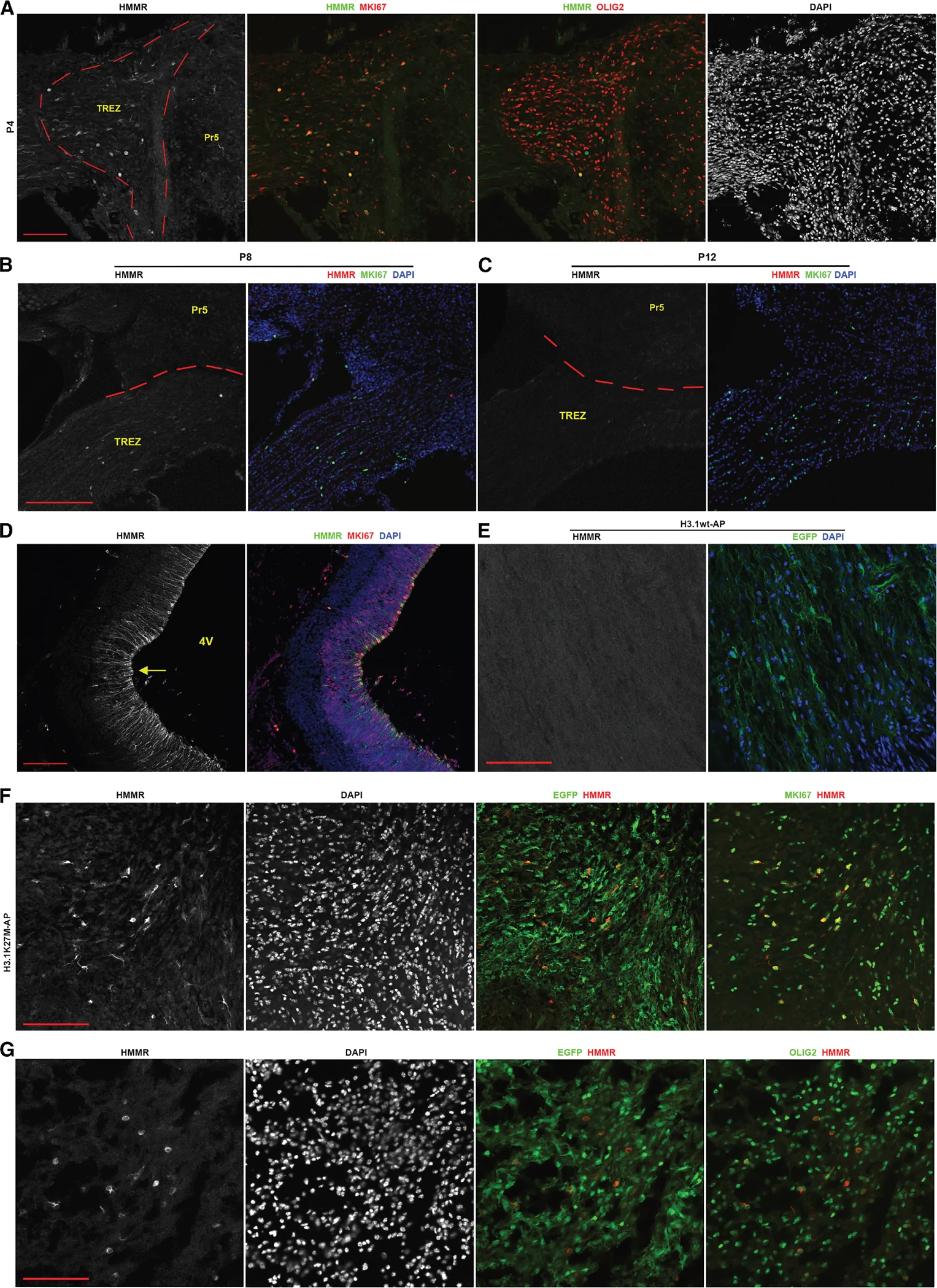
Expression of HMMR in the TREZ, NSCs, and glioma cells (A–C) Developmental expression of HMMR in the TREZ. Dashed lines outline the TREZ area in (A) and the approximate border between the TREZ and non-TREZ areas in (B) and (C). Images are representative of four samples for each group. Scale bars: 100 μm. (D) HMMR is expressed in NSCs in the embryonic mouse brainstem at E11.5. Cells with relatively high HMMR immunoreactivity appear to be bipolar and are localized at the ventricular zone (MKI67^+^ layer). This pattern is consistent with NSCs. The arrow indicates the pontine flexure. Scale bars: 100 μm. (E–G) HMMR is overexpressed in a subset of glioma cells transfected with H3.1K27M-AP. Images are representative of three or more samples for each group. Scale bars: 100 μm. Abbreviations are used as follows: 4 V, the fourth ventricle; Pr5, principal sensory nucleus of the trigeminal nerve. See also Figure S6.

The role of *Hmmr* in H3.1K27M-AP-mediated and TREZ-specific gliomagenesis was assessed in three independent ways. First, immunostaining experiments monitored HMMR expression in developing brainstem and in glioma cells transfected with H3.1K27M-AP. Increased HMMR expression was evident in the TREZ niche relative to non-TREZ areas during early postnatal stages (P4 and P8) (Figures 6A and 6B). Moreover, the TREZ is enriched with proliferating OLIG2-lineage cells at these early postnatal stages (Figures S6A–S6G). HMMR expression became undetectable at later postnatal stages (P12 and later) (Figure 6C). In embryonic stages, HMMR was specifically expressed in NSCs in the mouse brainstem at E11.5 (Figure 6D). That is, the stage when mutations were introduced into the brainstem in our mouse models. In glioma cells transfected with H3.1K27M-AP, a subset of cells expressed HMMR, and nearly all of these were proliferating OLIG2^+^ cells (Figures 6F and 6G). No HMMR immunoreactivity was detected in H3.1wt-AP control tissues (Figure 6E). Taken together, these expression data are consistent with HMMR involvement in TREZ-specific gliomagenesis induced by H3.1K27M-AP. We did detect a subset of transfected proliferating cells outside of the TREZ in mice of the H3.3K27M-TPC and D842V groups that also exhibited HMMR immunoreactivity (Figures S6H and S6I). That observation is consistent with HMMR expression promoting gliomagenesis in genetic contexts that do not require a TREZ niche for development.

**Figure 7.**
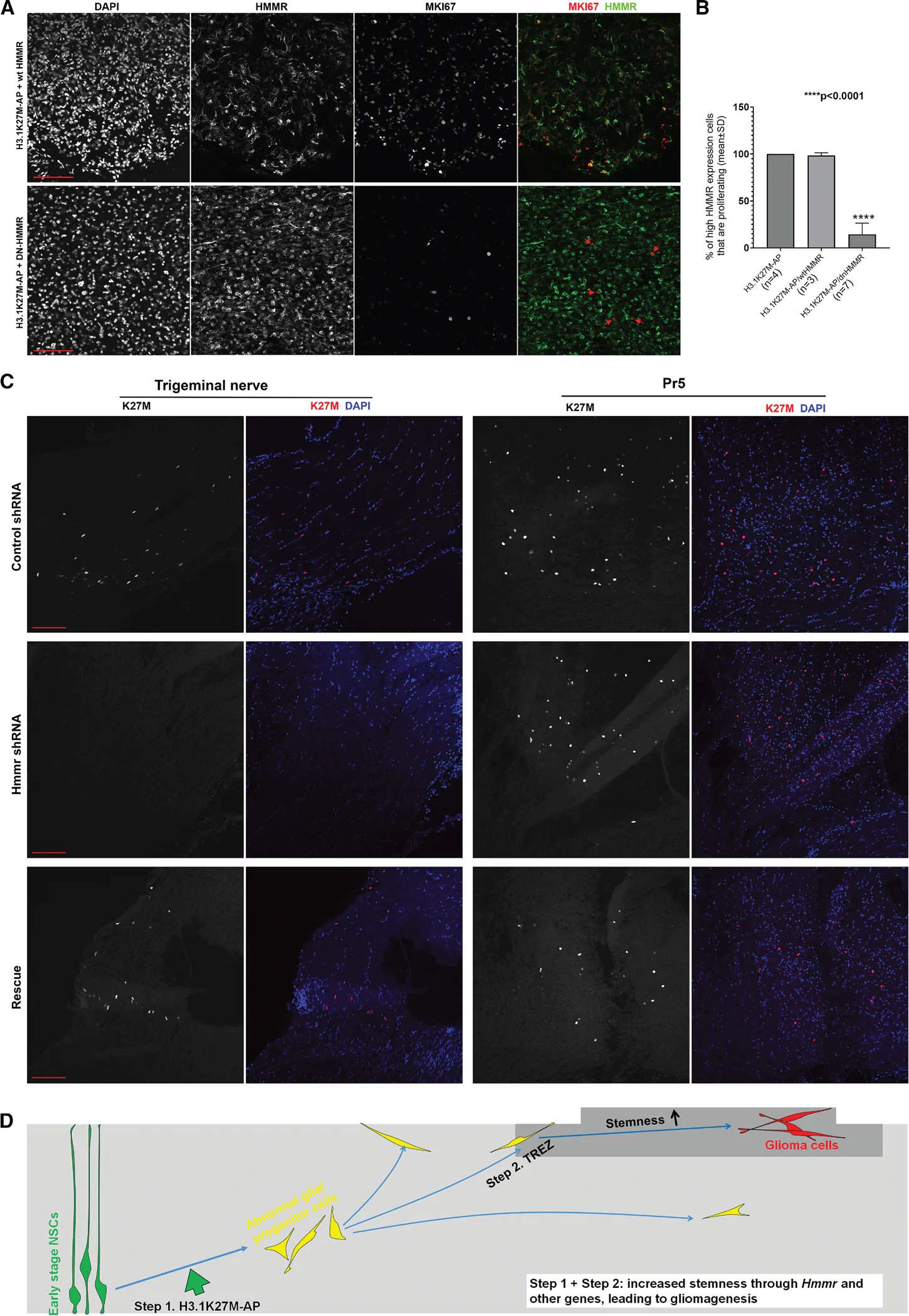
Role of HMMR in TREZ gliomagenesis induced by H3.1K27M-AP (A and B) A dominant-negative mutant of HMMR inhibits glioma cell proliferation induced by H3.1K27M-AP. (A) Confocal images from the trigeminal nerve of a P60 control mouse (H3.1K27M-AP co-transfected with wild-type HMMR) and a P55 DN-HMMR mouse (H3.1K27M-AP co-transfected with DN-HMMR) are shown. Images are representative of three control mice (P28–P61) and seven DN-HMMR mice (P21–P60). Scale bars: 100 μm. (B) Quantifications of the proliferation rate among cells with relatively high HMMR immunoreactivity (20–25 cells with the highest HMMR immunoreactivity in each confocal image frame were included). Transfected cells in the H3.1K27M-AP group that were not co-electroporated with an HMMR construct were also included in the quantifications. ****p < 0.0001, one-way ANOVA. (C) Knockdown of HMMR leads to the absence of H3.1K27M-AP cells on the trigeminal nerve. Images are representative of mice at the age of 1.5–3 months. Scale bars: 50 μm. (D) Model of TREZ gliomagenesis induced by H3.1K27M-AP. See also Figure S7.

To more directly determine whether HMMR supports pontine H3.1K27M-AP-induced gliomagenesis, a Tol2 transposon-based plasmid for expressing a dominant-negative HMMR (DN-HMMR) was co-electroporated with H3.1K27M-AP expression plasmids into the brainstem of E11.5 mouse embryos. DN-HMMR harbors C-terminal amino acid substitutions that abrogate hyaluronan binding and thereby render this a dominant-negative variant that inhibits hyaluronan signaling.^41^ Gliomagenesis in the brainstem of mice born from the electroporated embryos was subsequently analyzed. In the control group where DN-HMMR was replaced by wild-type HMMR, nearly all cells with high HMMR immunoreactivity were proliferative (Figures 7A and 7B). This pattern was indistinguishable from that recorded for mice transfected with H3.1K27M-AP alone (Figures 6F and 7B). In stark contrast, most cells of the DN-HMMR group with high HMMR immunoreactivity were not proliferative (Figures 7A and 7B). While gliomagenesis still occurred, cell proliferation was restricted to those cells with low HMMR immunoreactivity and low ectopic expression of DN-HMMR (Figure 7A). Those data demonstrate that interfering with HMMR activity inhibits pontine H3.1K27M-AP-induced glioma cell proliferation.

Third, a short hairpin RNA (shRNA) knockdown approach was performed to complement the DN-HMMR experiments. A transposon-based shRNA plasmid was constructed for silencing *Hmmr*. When introduced into the embryonic mouse brainstem at E11.5, this construct markedly reduced the fraction of transfected cells that exhibited HMMR immunoreactivity at E15.5 (Figures S6J and S6K). The *Hmmr* shRNA construct (or control shRNA construct) was co-electroporated with the H3.1K27M-AP plasmid mixture into the embryonic mouse brainstem at E11.5, and gliomagenesis was analyzed at postnatal stages. Unexpectedly, markedly fewer K27M^+^ cells (and EGFP^+^ cells) were observed in all samples, and no gliomagenesis was observed in any mice born from the electroporated embryos. This could be another example of non-specific gene silencing in persistent and long-term shRNA expression.^42,43^ Although gliomagenesis could not be assessed in these samples, an intriguing phenotype was observed in the *Hmmr* shRNA group. In both the control shRNA group (three mice) and the rescue group (three mice; a transposon-based plasmid for expressing shRNA-resistant wild-type HMMR was added to the *Hmmr* shRNA/H3.1K27M-AP plasmid mixture in this group), a subset of K27M^+^ cells was distributed to the trigeminal nerve (Figure 7C). No K27M^+^ cells were present on the trigeminal nerve in the *Hmmr* shRNA group (10 mice)—even though comparable numbers of K27M^+^ cells were observed at non-TREZ locations among these groups (Figure 7C). Marker analyses reported that the K27M^+^ cells on the trigeminal nerve were glial cells (Figure S7). This phenotype is consistent with a model in which HMMR supports the stemness/self-renewal of NSCs. As is the case in other brain regions, the brainstem undergoes neurogenesis first and gliogenesis later in development.^44–47^ Reduced stemness/self-renewal of NSCs would lead to premature depletion of NSCs during neurogenesis at earlier stages and consequently reduced gliogenesis at later stages. Other factors, such as increased cell death or altered cell specification or migration, might also contribute to the observed phenotype.

### *HMMR* expression in human pediatric brain tumors

To assess whether HMMR may play a role in human pediatric brain tumors, we inspected available *HMMR* expression profiles in human pediatric brain tumor databases. Examination of the PeCan database^48^ confirmed expression of *HMMR* mRNA in DMGs, H3K27-altered carrying an *H3.1K27* mutation (Figure S7G). Furthermore, search of the PedcBioPortal database^49–51^ confirmed expression of *HMMR* mRNA in pediatric patients with DMGs harboring co-occurring *H3.1K27M*/activating *ACVR1* mutations (Figure S7H). Comparisons of pediatric brain tumors without co-occurring *H3.1K27M*/activating *ACVR1* mutations (952 patients with high *HMMR* mRNA and 841 patients with unaltered *HMMR* mRNA relative to healthy tissues) to pediatric brain tumors with co-occurring *H3.1K27M*/activating *ACVR1* mutations (all DMGs, H3K27-altered; 8 patients with high *HMMR* mRNA and 1 patient with unaltered *HMMR* mRNA relative to healthy tissues) reported that the latter group was more likely to display high *HMMR* mRNA (Fisher’s exact test, two-sided: *p* value = 0.0422). These database results provide independent support for a role for HMMR signaling in human pediatric brain tumors harboring co-occurring *H3.1K27M*/activating *ACVR1* mutations.

## DISCUSSION

Our discoveries provide a physiological basis for the long-standing puzzle for why the *H3.1K27M*/activating *ACVR1* mutation pair is featured in pediatric DIPGs but not in pediatric HGGs that arise from other anatomical locations. As illustrated in Figure 7D, we propose that the genetic/anatomical specificity of this subset of pediatric DIPGs rests on two primary principles: (1) the large axon tracts of the TREZ provide a uniquely supportive niche for persistent glioma cell proliferation, and (2) H3.1K27M-AP enhances oligodendrocyte-lineage stem/progenitor cell stemness and drives persistent proliferation of these stem-like cells.

### The unique role of the TREZ in DIPGs

We demonstrate that introduction of DIPG-specific driver mutations into the mouse brain at E11.5 recapitulates the signature anatomical specificities of driver mutations in pediatric HGG patients. A striking feature of the rapid *de novo* gliomagenesis induced by H3.1K27M, ACVR1-G356D, and PIK3CA-E545K (i.e., H3.1K27M-AP) co-expression is a restriction of glioma cell proliferation to the TREZ. Previous studies demonstrate an involvement of the trigeminal nerve in a subset of DIPG patients^52^ and report that neuronal activities stimulate gliomagenesis.^32–34^ Our collective data, when coupled with those observations, suggest why the TREZ uniquely supports gliomagenesis induced by *H3.1K27M* and its common co-occurring DIPG mutations. The TREZ is anatomically unique in that several large, myelinated axon tracts intersect in this area, including the trigeminal nerve, the middle cerebellar peduncle, and the trapezoid body.^53–56^ Thus, it is possible that the activities of these axon tracts contribute to gliomagenesis. Interestingly, these activities can be tuned noninvasively.^57–60^ These capabilities raise the possibility that *H3.1K27M*/*ACVR1*-dependent DIPGs will be responsive to appropriate activity modulation of these neuronal systems.

### A biochemical basis for the uniquely supportive niche provided by the TREZ

The high myelination demand of the large axon tracts in the TREZ likely drives the persistent proliferation of oligodendrocyte-lineage stem/progenitor cells whose stemness is enhanced by H3.1K27M-AP. Hyaluronan is an ECM glycosaminoglycan that mediates the interaction between neurons and oligodendrocyte-lineage cells during myelination.^31^ This function identified its receptor HMMR as an attractive candidate for participating in such a cell growth control circuit. Our data reinforce this idea. HMMR is highly expressed in the TREZ during development and gliomagenesis. Moreover, interfering with HMMR activity markedly impairs H3.1K27M-AP-induced glioma cell proliferation. Results from *Hmmr* knockdown experiments lend further evidence that HMMR contributes to maintenance of H3.1K27M-AP cell stemness. Nonetheless, interpretation of the shRNA data is complicated by our observation that some generalized gene silencing effects occurred—possibly due to persistent long-term shRNA expression. This limitation can be addressed by future experiments using more involved approaches that are unlikely to induce such generalized silencing effects. These might include inducible or post-initiation knockdown strategies and/or knockout mice.

Although HMMR is also implicated in mitotic spindle positioning and cell cycle progression,^35,36^ the defects in gliomagenesis documented herein unlikely reflect indirect effects of deranged cell cycle progression. Analyses of two different lines of *Hmmr* knockout mice revealed that neither absence of HMMR nor deletion of the HMMR C terminus compromises cell cycle progression in the brain—even though both lesions compromise NSC self-renewal.^39,40^

### Developmental origin of DIPGs

Oligodendrocyte precursor cells (OPCs) or OPC-like cells are proposed to be the origin of DIPGs.^14,18,19^ Moreover, cell stemness plays a key role in DIPG pathogenesis.^11,16,17^ In our study, while the tumor cells were characterized by expression of oligodendrocyte-lineage markers, the tumor initiation event occurred in early-stage NSCs. Thus, our data are generally consistent with previous findings that both cell stemness and oligodendrocyte-lineage developmental program play important roles in the origin of DIPGs. While DIPG cases typically display astrocytoma histology,^61^ most proliferating cells in these astrocytic tumors express the oligodendrocyte-lineage marker OLIG2.^62^ We therefore find it significant that the tumor tissues induced by H3.1K27M-AP overexpressed the astrocytic marker GFAP, and most of the proliferating cells in tumor tissues scored as OLIG2^+^. Our data also provide an interesting perspective on previous suggestions that DIPGs originate from a Hedgehog-responsive progenitor cell population at the ventral pons.^63^ The anatomical location of this suspect cell population is adjacent to the TREZ. Whether this population overlaps with those that give rise to TREZ-specific gliomagenesis merits future investigation.

### Why does our experimental model faithfully reproduce the anatomical specificities of human disease whereas previous GEMMs do not?

A major distinction between our experimental strategy and previous GEMMs is the embryonic stage at which the gliomagenic mutations are introduced. Previous GEMM studies introduced/expressed the driver mutations in the brainstem at: (1) E12.5, (2) at the neonatal stage, or (3) in OLIG2^+^ progenitors and their progeny.^13–15^ Our experimental approach introduces the genetic elements into the brainstem at an earlier stage where stem/progenitor cells do not yet express OLIG2 (E11.5). We consider this a highly significant matter given cell stemness is a critical factor in DIPG pathogenesis. Why does our model not reproduce the development of gliomas in other brain regions? We suggest this to be a simple consequence of the rampant H3.1K27M-AP-induced gliomagenesis in the TREZ resulting in early mortality that precedes the development of gliomas in other brain regions.

### Limitations of the study

The limitations are severalfold. First, we do not yet understand how neuron-glioma interactions act through HMMR to promote gliomagenesis induced by H3.1K27M-AP. The overexpression of HMMR in the H3.3K27M-TPC and D842V samples suggests that, while important for supporting TREZ gliomagenesis induced by H3.1K27M-AP, HMMR does not by itself represent a TREZ-specific signaling pathway. Such TREZ-specific signals remain to be identified. Second, the data reported herein focus on H3.1K27M-AP and its co-associated DIPG mutations. Demonstration that H3.3K27M-TPC-induced glioma-like cell proliferation is stimulated by different neuron-glia interactions or electrical stimuli would expand the generality of the developmental and biochemical concepts outlined herein. Third, it remains to be determined how accurately co-introducing driver mutations into the mouse brain at E11.5 recapitulates the timing of driver mutation engagement in human patients.

## RESOURCE AVAILABILITY

### Lead contact

Further information and requests for resources and reagents should be directed to and will be fulfilled by the lead contact, Zhigang Xie (zxie@tamu.edu).

### Materials availability

Plasmids generated in this study will be available upon request.

### Data and code availability

- All data will be provided by the lead contact upon request.
- This paper does not report original code.
- Any additional information required to reanalyze the data reported in this work is available from the lead contact upon request.

## STAR★METHODS

### EXPERIMENTAL MODEL AND STUDY PARTICIPANT DETAILS

#### Mouse models

The mice used in this study are on the C57BL6 background. These mice are maintained in the animal facility of Texas A&M University Health Science Center and handled in accordance with National Institutional Health and institutional guidelines on the care and use of animals. The animal use protocol numbers approved by the Institutional Animal Care and Use Committee (IACUC) of Texas A&M University Health Science Center for this study are 2017–0130, 2019–0087, 2022–0045, 2023–0054, and 2026–0039, covering consecutive active periods from 2017 to the present.

### METHOD DETAILS

#### Plasmids

Transposon-based plasmids for expressing wild-type or mutant proteins were generated using the Tol2 transposon system.^22^ A 4.0kb fragment carrying the coding sequence for Cre:GFP flanked with a CAG promoter and a rabbit globin polyA sequence was cut from pCAG-Cre:GFP^65^ with SpeI/HindIII and inserted into the XbaI/HindIII sites of pMiniTol2.^24^ The 1.8kb EcoRI/NotI fragment of the insert containing the Cre:GFP coding sequence was then replaced with a 0.7kb EcoRI/NotI fragment containing EGFP coding sequence obtained via polymerase chain reaction (PCR) using pEGFPc1 (Clontech) as the template. In the resulting plasmid (referred to as pMiniT2-EGFP), the EGFP expression cassette (CAG promoter, EGFP coding sequence, and rabbit globin polyA sequence) was flanked by Tol2 inverted terminal repeats (ITRs).

To construct transposon plasmids for expressing glioma-related proteins, coding sequences for these proteins were amplified via reverse transcription (RT)-PCR using complementary DNA (cDNA) prepared from an E14.5 mouse embryonic brain, and used to replace the EGFP coding sequence of pMiniT2-EGFP. These sequences include *Hist1h3i* (encoding H3.1), *H3f3a* (encoding H3.3), *Acvr1*, *Pik3ca* (encoding the catalytic subunit of phosphoinositide-3-kinase), *Trp53*, *Pdgfra*, *Ccnd2*, and *Hmmr*. Note that, while in humans both *HIST1H3B* and *HIST1H3I* encode H3.1, in mice only *Hist1h3i* encodes H3.1 (mouse *Hist1h3b* encodes H3.2). For expressing mutant proteins H3.1-K27M, H3.3-K27M, ACVR1-G356D, PIK3CA-E545K, TRP53-R172H, PDGFRA-D842V, and DN-HMMR (which harbors the mutations K723E, K727E, K748N, R749W, and K750E), site-directed mutagenesis was performed to introduce the corresponding mutation into the coding sequences. Both wild-type and mutant coding sequences were verified by sequencing. The transposon-based shRNA plasmid for silencing the mouse *Hmmr* gene was constructed by inserting an shRNA expression cassette (comprised of U6 promoter, small hairpin sequence targeting *Hmmr*, and poly T) into the EcoRI and HindIII sites of pMini-Tol2. The *Hmmr* target sequence was: GGCACAGTCACAAACAATTAA (located at the 3′-UTR of the transcript). For the control shRNA, the *Hmmr* small hairpin sequence of the *Hmmr* shRNA plasmid was replaced with a random sequence small hairpin that does not target any mouse genes.

#### Animals and *in utero* electroporation

Mice were on the background of C57BL6, and were maintained in the animal facility of Texas A&M University Health Science Center. All animal work was performed according to protocols approved by the Institutional Animal Care and Use Committee (IACUC) of Texas A&M University and the animal use guidelines of the National Institute of Health. The *in utero* electroporation procedure was performed similar to that described in our previous studies with several modifications (e.g., on the site of DNA injection and the orientation of the electrodes).^64,66,67^ Briefly, mouse breeding was set up to produce embryos at the stage of E11.5. The date of plug observation was designated as E0.5. At the stage of E11.5, the pregnant mouse was anesthetized and placed onto a surgical platform. An abdominal incision at the size of about 1 centimeter was made, and the uteri were gently pulled out of the abdominal cavity. For brainstem transfection, a mixture of the DNA solution (prepared in sterile water, mixed with ~0.05% Fast Green dye) was injected into the fourth ventricle via a sterile glass micropipette, which were inserted into the aqueduct with the tip pointing toward the fourth ventricle. This method of injection ensured that the brainstem area to be transfected would not be disrupted mechanically by micropipette insertion. Following DNA solution injection, five electric pulses (~17 volts; 50msec on/950msec off; generated from an ECM830 square wave electroporator) were delivered across the mouse embryo via tweezer-like disc electrodes. During the delivery of the electric pulses, the negative electrode disc was positioned on the back of the head of the mouse embryo (the direct contact was on the uterine wall), and the positive electrode disc was positioned at tail side of the embryo. By this positioning, the electric pulses were expected to be delivered across the pontine flexure. For forebrain/midbrain electroporation, the DNA solution was injected from the right lateral ventricle into the lateral ventricles, the third ventricle, and the aqueduct. After DNA injection, the electrode discs were placed on opposite sides of the embryonic head so that delivery of the electric pulses would result in transfection of cells at the cerebral cortex (where cells took up the plasmids from the lateral ventricle), the thalamus (where cells took up the plasmids from the third ventricle), and the midbrain (where cells took up the plasmids from the aqueduct). After the delivery of the electric pulses, the uteri were placed back into the original position in the abdominal cavity of the dam, the incision was sutured, and the dam was placed in a warm location for recovery. Aseptic handling, analgesics (pre- and post-operation), and post-operational monitoring were used according to the approved methods by the IACUC of Texas A&M University. The electroporated embryos were allowed to develop to term, and euthanized at different postnatal or adult stages.

#### Brain sample collection and processing

A subset of mice born from electroporated embryos developed hydrocephalus at different postnatal stages. These mice were promptly euthanized for brain harvest when the sign of hydrocephalus (i.e., domed head) was observed. A subset of mice expressing H3.1K27M-AP displayed head-tilting phenotype. These mice were also promptly euthanized for brain harvest upon occurrence of the head-tilting phenotype. A subset of mice were also euthanized for brain harvest without the occurrence of any symptoms. For brainstem transfected mice, the whole brain was dissected out upon euthanasia, and divided into two-halves by a sagittal midline cut. The forebrain hemispheres were then removed, and the remaining part of the samples (including the brainstem and the cerebellum) were examined for EGFP fluorescence under an epifluorescence inverted microscope (Nikon Eclipse TS100) with a 10× objective. To minimize the effects of transfection locations (e.g., failure to transfect a particular brainstem region) on the specificity of anatomical locations of gliomagenesis, only the samples with clearly visible green fluorescence on both the medial side of the brainstem and the lateral side of the brainstem were collected for further processing and analysis.

For forebrain/midbrain transfected mice, the whole brain was dissected out upon euthanasia, and divided into two-halves by a sagittal midline cut. Both halves of the samples were checked for EGFP fluorescence under an epifluorescence inverted microscope (Nikon Eclipse TS100) with a 10× objective. The sample halves with clearly visible green fluorescence on the cerebral cortex and/or the thalamus/midbrain areas were collected for further processing and analysis.

For cryosection preparation, the collected samples were fixed in 2% paraformaldehyde prepared in phosphate buffered saline (PBS) at room temperature for 45 min, soaked in 20% sucrose prepared in PBS until the samples sank, and embedded in Tissue-Tek O.C.T. The embedded samples were stored at −20°C until cryosection preparation. Sagittal sections (20μm) were prepared for brainstem transfected samples, and either sagittal or coronal sections (20μm) were prepared for forebrain/midbrain transfected samples. For paraffin section preparation, the collected samples were fixed in 10% neutral buffered formalin for 48 h. Paraffin section (4μm) preparation and H&E staining were performed by the Veterinary Medicine & Biomedical Sciences Core Histology Lab at Texas A&M University.

#### Antibodies and fluorescent immunostaining

Primary antibodies are listed in the key resources table. Secondary antibodies (Jackson Immunoresearch) were conjugated with cyanine dyes and were with minimal cross reactivity. The cryosections were incubated in the primary antibodies prepared in PBS containing 3% bovine serum albumin (BSA) and 0.2% Triton X-100 at room temperature overnight, secondary antibodies prepared in PBS containing 3% BSA and 0.2% Triton X-100 at room temperature for one hour, and DAPI solution (5μg/mL in PBS) for 5min. After staining, EverBrite Hardset mounting medium (Biotium) was used to mount coverslips.

#### Confocal microscopy and imaging processing

Images of immunostained brain sections were acquired on a Nikon confocal microscope controlled by the NIS-Elements software. For some of the analyses, confocal images of different regions of the same section were stitched together using the ImageJ software.

#### Identification of anatomical locations in the mouse brain

The anatomical locations of the brainstem were identified by comparing the pattern of DAPI staining to the pattern of Nissl staining and acetylcholinesterase staining in published mouse brain atlases.^55,56^ Several major axon tracts in the brainstem (such as the trigeminal nerve, the spinal trigeminal tract, the middle cerebellar peduncle, the inferior cerebellar peduncle, and the trapezoid body) revealed by Nissl staining and acetylcholinesterase staining in the brain atlases^55,56^ can be identified in the corresponding regions within the DAPI stained sections based on the special arrangement of the DAPI-labeled cell nuclei - the cell nuclei on axon tracts are arranged as aligned strings.

#### Search of human pediatric brain cancer databases

We searched the St. Jude PeCan database^48^ and the PedcBioPortal database^49–51^ on the date of June 5, 2026 to assess the expression of *HMMR*. For the PeCan database search, the parameters Brain Tumor (Subtype Root), DMGH3K27 (Subtype), *HIST1H3B K28* (Subtype Biomarker), and *HMMR* (Gene) were used. For the PedcBioPortal search, the Pediatric Brain Tumor Atlas (PBTA, Provisional) dataset was selected as the Study, mRNA expression/RNA expression z-scores vs. healthy was selected as the Genomic Profile, and *H3C2* (which encodes the H3.1 protein), *ACVR1*, and *HMMR* were selected as queried genes.

### QUANTIFICATION AND STATISTICAL ANALYSIS

Statistical analyses were performed using the GraphPad Prism (version 10) software. Student’s *t* test (when variances were equal) or Welch’s *t* test (when variances were not equal) was used to determine the significance of the differences between two groups. One-way ANOVA was used to determine the significance of the differences among three or more groups based on one factor, and two-way ANOVA was used to determine the effects of two factors (e.g., genetic mutation and anatomical location) and their interactions on a dependent variable (e.g., cell proliferation). Log rank test was used to compare different groups in the survival analysis. Fisher-Freeman-Halton exact test (available at http://vassarstats.net; accessed March 31,2026) was used to compare the frequency of hydrocephalus among the H3.1K27M-AP group and the control groups. Fisher’s exact test was used to compare the frequency of *HMMR* high mRNA between pediatric brain tumors with and without co-occurring *H3.1K27M*/activating *ACVR1* mutations.

## Supplementary Material

1

2

SUPPLEMENTAL INFORMATION

Supplementary data related to this article can be found online at https://doi.org/10.1016/j.celrep.2026.117796.

## Figures and Tables

**KEY RESOURCES TABLE:** 

REAGENT or RESOURCE	SOURCE	IDENTIFIER

Antibodies		

Chicken polyclonal anti-GFP	Aves Labs	Cat#GFP-1020; RRID: AB_10000240
Goat polyclonal anti-GFP	Novus Biologicals	Cat# NB100-1770; RRID:AB_10128178
Rabbit polyclonal anti-MKI67	Abcam	Cat# ab15580; RRID:AB_443209
Rat monoclonal anti-MKI67	Invitrogen	Cat#14-5698-82; RRID:AB_10854564
Goat polyclonal anti-OLIG2	R&D Systems	Cat# AF2418; RRID:AB_2157554
Rabbit monoclonal anti-H3-K27M	Abcam	Cat# ab190631; RRID:AB_2860570
Rabbit monoclonal anti-HMMR	Abcam	Cat# ab124729; RRID:AB_10975797
Rabbit monoclonal anti-PDGFRA	Cell Signaling Technology	Cat# 3174; RRID:AB_2162345
Chicken polyclonal anti-GFAP	Aves Labs	Cat# GFAP; RRID:AB_2313547
Rat monoclonal anti-NESTIN	Abcam	Cat# ab81462; RRID:AB_1640724
Rabbit monoclonal anti-ASCL1	Abcam	Cat# ab211327; RRID:AB_2924270
Rat monoclonal anti-histone H3 phospho-Ser28	Abcam	Cat# ab10543; RRID:AB_2295065
Rabbit monoclonal anti-phospho-SMAD1/SMAD5/SMAD9	Cell Signaling Technology	Cat#13820 S; RRID:AB_2493181
Rabbit monoclonal anti-phospho-AKT (Ser473)	Cell Signaling Technology	Cat#4060 S; RRID:AB_2315049
Rabbit monoclonal anti-H3K27me3	Cell Signaling Technology	Cat#9733 S; RRID: AB_2616029
Rat monoclonal anti-CD45	Novus Biologicals	Cat#NB100-77417; RRID: AB_1083776

Chemicals, peptides, and recombinant proteins		

Agarose LE, quick dissolve	Genesee Scientific	Cat#9012-36-6
Bovine serum albumin	EMD Millipore Corp	Cat#126575
DAPI	Biotium	Cat#40011
EverBrite Hardset Mounting Medium	Biotium	Cat#23003
Paraformaldehyde, aqueous solution	Electron Microscopy Sciences	Cat#15710
10% Neutral Buffered Formalin concentrate	StatLab	Cat#BF128
Phosphate buffered saline, 1× sterile solution	Dot Scientific Inc	Cat#DSP10400-1000
Sucrose	Sigma	Cat#S7903

Critical commercial assays		

iScript select cDNA synthesis kit	Bio-Rad	Cat#1708897
Phusion high-fidelity DNA polymerase	New England BioLabs	Cat#M0530L
Plasmid DNA minipreps kit	Bio Basic	Cat#BS614-250Preps
T4 DNA ligase	New England BioLabs	Cat#M0202L
Tissue-Tek O.C.T. compound	Sakura	Cat#4583

Experimental models: Organisms/strains		

C57BL6 mice	Xie et al.^64^	N/A

Recombinant DNA		

pCAG-Tol2	This study	N/A
pMiniTol2-EGFP	This study	N/A
pMiniTol2-H3.1K27M	This study	N/A
pMiniTol2-H3.1wt	This study	N/A
pMiniTol2-ACVR1-G356D	This study	N/A
pMiniTol2-PIK3CA-E545K	This study	N/A
pMiniTol2-H3.3K27M	This study	N/A
pMiniTol2-H3.3wt	This study	N/A
pMiniTol2-TRP53-R172H	This study	N/A
pMiniTol2-PDGFRA	This study	N/A
pMiniTol2-PDGFRA-D842V	This study	N/A
pMiniTol2-CCND2	This study	N/A
pMiniTol2-DN-HMMR	This study	N/A
pMiniTol2-HMMR	This study	N/A
pMiniTol2-U6-*Hmmr* shRNA	This study	N/A
pMiniTol2-U6-control shRNA	This study	N/A

Software and algorithms		

NIS Elements (version 5.21.00)	Nikon	N/A
ImageJ (version 1.54f)	Rasband, W.S., U.S. National Institute of Health	N/A
GraphPad Prism (version 10.1.2)	GraphPad Software, Inc	N/A

## Highlights

- Diffuse intrinsic pontine glioma mutations are introduced into mouse embryo brains
- Transfected neural stem cells generate cells with broad distribution in the brain
- *De novo* gliomagenesis is induced specifically in the trigeminal root entry zone
- HMMR is important for gliomagenesis in the trigeminal root entry zone
