# Supplementary material for: A pontine-specific niche supports de novo gliomagenesis
Source: Cell Rep. Author manuscript; Available in PMC 2026 Sep 18. (PMC13587746; doi:10.1016/j.celrep.2026.117796)
Supplement: Supplementary file 1 [file NIHMS2206907-supplement-1.pdf]

Cell Reports, Volume *45*

Supplemental information

A pontine-specific niche supports *de novo* gliomagenesis

Adrija Pathak, Gregg B. Wells, Vytas A. Bankaitis, and Zhigang Xie

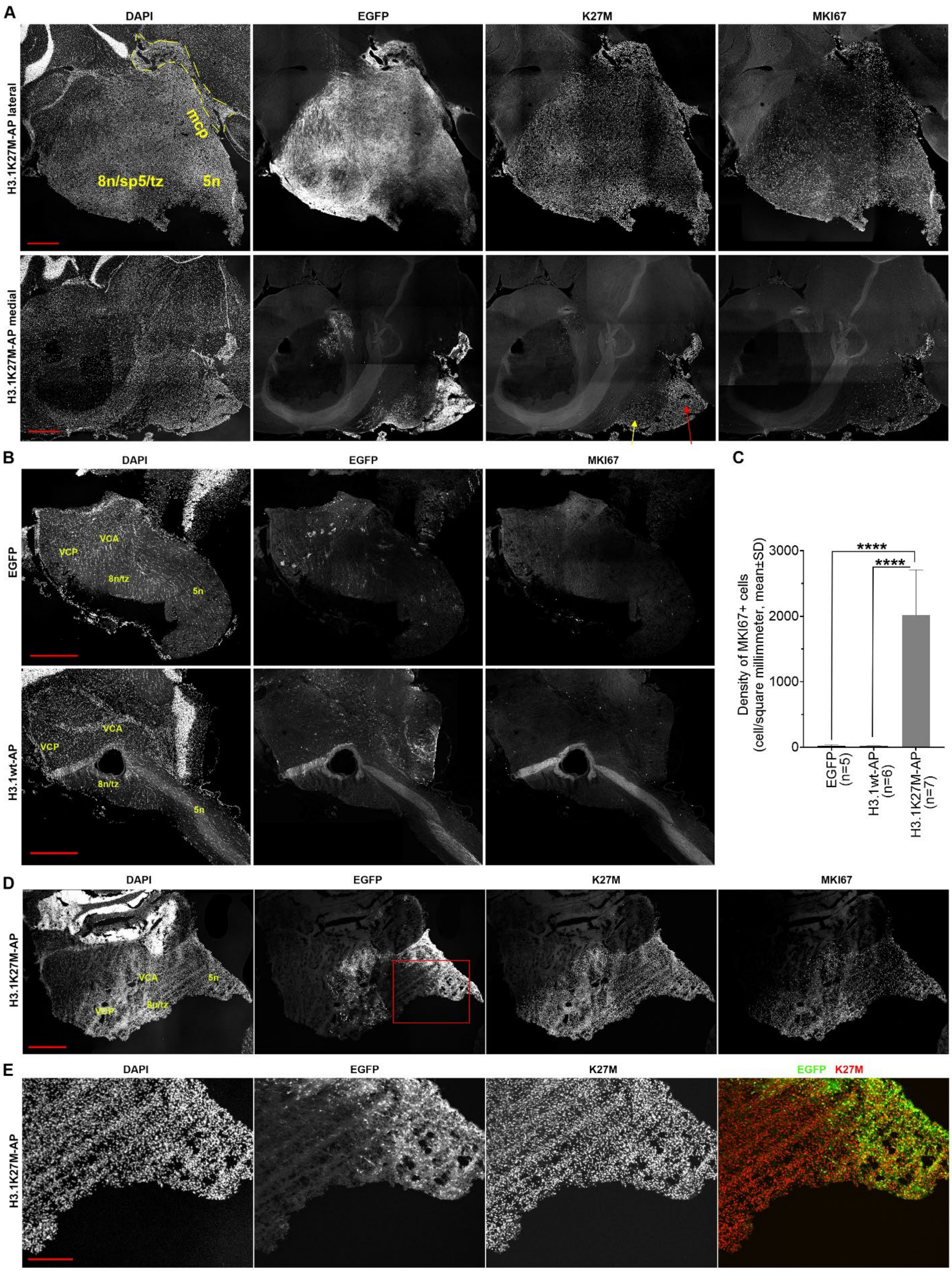

A
DAPI
EGFP
K27M
MKI67
H3.1K27M-AP lateral
mcp
8n/sp5/tz
5n
H3.1K27M-AP medial
B
DAPI
EGFP
MKI67
EGFP
VCP
VCA
8n/tz
5n
H3.1wt-AP
C
Density of MKI67+ cells (cell/square millimeter, mean±SD)
3000
2000
1000
0
****
****
EGFP (n=5)
H3.1wt-AP (n=6)
H3.1K27M-AP (n=7)
D
H3.1K27M-AP
E
EGFP K27M

Figure S1. Expression of H3.1K27M-AP leads to de novo formation of high-grade tumors at the TREZ, related to Figure 1

(A) Images of the brainstem of a P52 mouse in the H3.1K27M-AP group. Composite confocal images of the lateral (upper panels) and medial (lower panels) sagittal sections of the brainstem are shown. $K27M^{+}MKI67^{+}$ tumor cells were detected throughout the lateral brainstem with the highest density around the TREZ and the brainstem surface (outlined by dashed lines). In the medial brainstem, $K27M^{+}MKI67^{+}$ tumor cells were limited to the surface of the ventral pons (red arrow) and at the medial part of the middle cerebellar peduncle tract (yellow arrow).

(B and C) Co-expression of wild-type H3.1 with activated ACVR1 and PIK3CA does not induce glioma-like growth in the mouse brainstem. Few proliferating cells are present in the brainstem of mice expressing EGFP alone or EGFP together with wild-type H3.1, ACVR1-G356D, and PIK3CA-E545K (i.e. H3.1wt-AP). Composite images of a P135 mouse of the EGFP group (upper panels) and a P74 mouse of the H3.1wt-AP group (lower panels) are shown. Quantifications of the number of proliferating cells in transfected areas of the brainstem are shown in **(C)**. Both $EGFP^{+}$ cells and $EGFP^{-}$ cells in transfected areas were counted. ****$p<0.0001$, one-way ANOVA. The n values represent the number of mice used for quantification in each group. Scale bars: 500μm.

(D and E) Not all transfected cells can be identified by EGFP in mice born from electroporated embryos. Composite confocal images of a P43 mouse in the H3.1K27M-AP group showing that many cells in this sample expressed the H3.1-K27M protein but not EGFP. The boxed area in (D) is shown in (E) at a higher magnification. Note that in (E) most tumor cells on the left side of the panel expressed H3.1-K27M but not EGFP. Scale bars: 500μm in (D) and 200μm in (E).

Abbreviations are used as follows. 5n: trigeminal nerve; 8n: the 8th cranial nerve; mcp: middle cerebellar peduncle; sp5: spinal trigeminal tract; tz: trapezoid body; VCA: ventral cochlear nucleus, anterior; VCP: ventral cochlear nucleus, posterior.

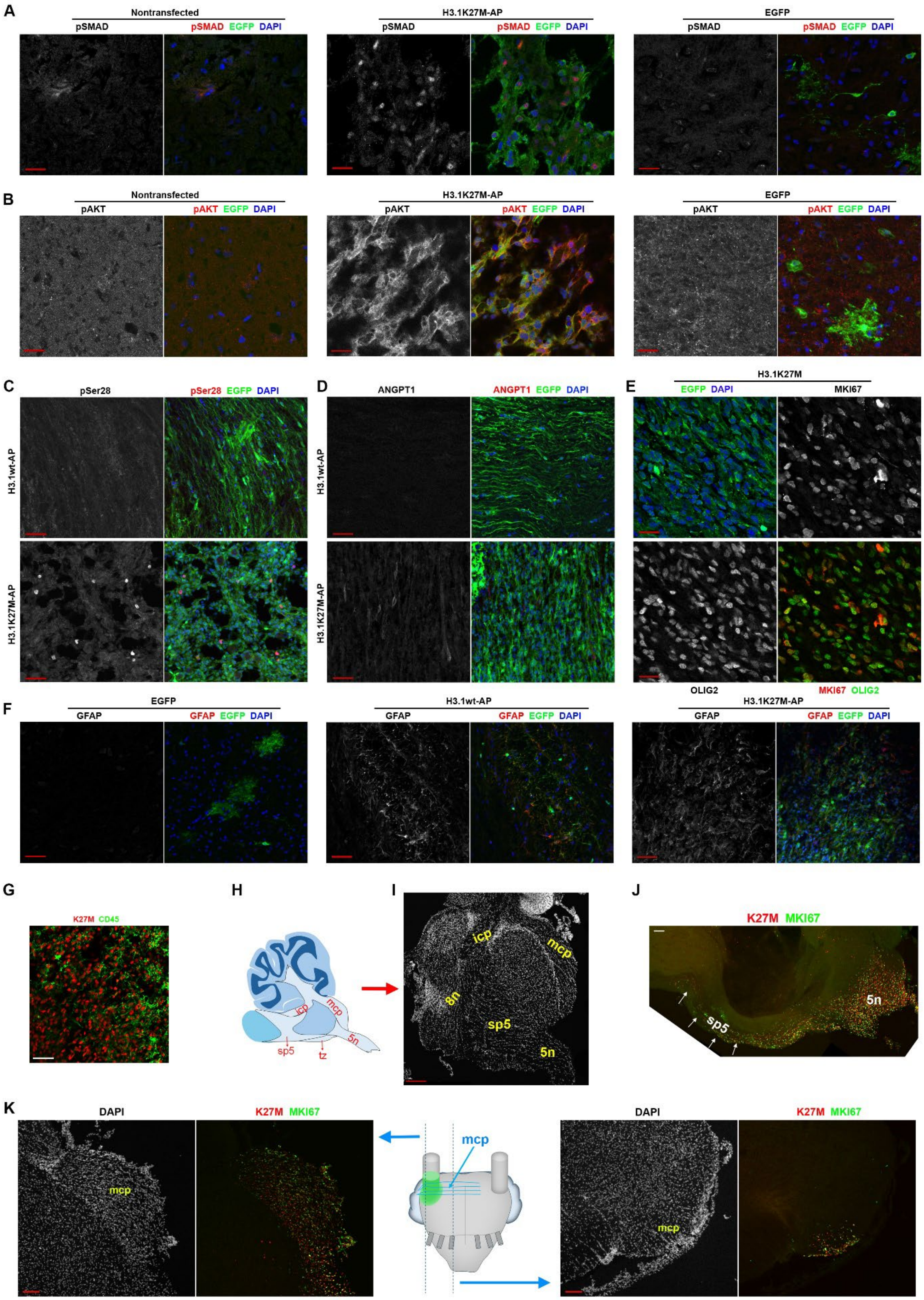
A
Nontransfected
pSMAD
pSMAD EGFP DAPI
H3.1K27M-AP
pSMAD
pSMAD EGFP DAPI
EGFP
pSMAD
pSMAD EGFP DAPI
B
Nontransfected
pAKT
pAKT EGFP DAPI
H3.1K27M-AP
pAKT
pAKT EGFP DAPI
EGFP
pAKT
pAKT EGFP DAPI
C
pSer28
pSer28 EGFP DAPI
H3.1wt-AP
H3.1K27M-AP
D
ANGPT1
ANGPT1 EGFP DAPI
H3.1wt-AP
H3.1K27M-AP
E
H3.1K27M
EGFP DAPI
MKI67
OLIG2
MKI67 OLIG2
F
EGFP
GFAP
GFAP EGFP DAPI
H3.1wt-AP
GFAP
GFAP EGFP DAPI
H3.1K27M-AP
GFAP
GFAP EGFP DAPI
G
K27M CD45
H
icp
mcp
5n
sp5
tz
I
icp
mcp
8n
sp5
5n
J
K27M MKI67
sp5
5n
K
DAPI
K27M MKI67
mcp
mcp
DAPI
K27M MKI67
mcp

Figure S2. Characterization of the tumors induced by H3.1K27M-AP, related to Figures 2 and 3

Cryosections of H3.1K27M tumor samples and control samples (EGFP and H3.1wt-AP; from ventrolateral pontine areas; corresponding to the site of tumorigenesis in H3.1K27M-AP samples) were immunostained and analyzed by confocal imaging to assess gene expression. Images are representative of three or more mice at the age of 1.5-3 months for each group.

(A) Assessment of ACVR1 activation using an antibody against phospho-SMAD1/SMAD5/SMAD9. Compared to non-transfected cells and cells transfected with EGFP, cells transfected with H3.1K27M-AP exhibited increased nuclear labeling by this antibody. Scale bars: 20μm.

(B) Assessment of phosphoinositide 3-kinase (PI3K) activity using an antibody against phospho-AKT1. Compared to non-transfected cells and cells transfected with EGFP, cells transfected with H3.1K27M-AP exhibited increased phospho-AKT1 immunoreactivity. Scale bars: 20μm.

(**C**) Detection of mitotic cells using an antibody against Serine 28-phosphorylated histone H3 (pSer28). Mitotic cells are present in H3.1K27M-AP tumor samples at high frequency but rarely detected in tissue samples transfected with H3.1wt-AP. Scale bars: 20μm.

(D) Expression of ANGPT1, a marker for angiogenesis, is increased in the H3.1K27M-AP tumor sample. No ANGPT1 immunoreactivity was detected in control samples transfected with H3.1wt-AP. Scale bars: 20μm.

(E) Co-immunostaining analyses of H3.1K27M-AP tumor samples show that most proliferating cells ($MKI67^+$) are $OLIG2^+$. Scale bars: 10μm.

(F) GFAP expression in control and tumor tissues. Compared to EGFP alone controls, samples transfected with H3.1K27M-AP or with H3.1wt-AP exhibited increased expression of GFAP. Scale bars: 20μm.

(G) The H3.1K27M-AP tumors are extensively infiltrated with myeloid cells. A P43 tumor sample was immunostained using an antibody against the myeloid marker CD45. Myeloid cells ($CD45^+$) cells were detected throughout the tumor mass. Scale bar: 20μm.

(H and I) Identification of anatomical locations in the mouse brainstem based on DAPI staining. Schematic illustrating the anatomical locations in sagittal sections of the mouse brainstem is shown in (H). Composite confocal images showing a DAPI stained lateral brainstem sagittal section prepared from a P20 mouse are shown in (I). Large axon tracts are characterized by nuclei arranged in strings that are generally aligned. These nuclei belong to oligodendrocytes that form the myelin sheath around the axons. The axon tracts identified based on the arrangement of DAPI-labelled nuclei are consistent with those identified based on Nissl staining and acetylcholinesterase staining[51,52]. Scale bar: 200μm.

(J and K) Proliferating cells expressing H3.1K27M-AP are present on axon tracts away from but directly linked to the TREZ. Composite confocal images of the brainstem of a P22 mouse expressing H3.1K27M-AP are shown in (J). Arrows indicate the part of the spinal trigeminal tract (sp5) relatively far away from the entry point of the trigeminal nerve (5n). Confocal images of the middle cerebellar peduncle (mcp) area in the brainstem of a P29 mouse expressing H3.1K27M-AP are shown in (K). Left panels show mcp at lateral pons where it intersects with the trigeminal nerve, right panels show mcp at a more medial side of the pons. Images are representative of eight mice expressing H3.1K27M-AP for both (J) and (K). Scale bars: 100μm.

Abbreviations are used as follows. 5n: trigeminal nerve; 8n: the 8th cranial nerve; icp: inferior cerebellar peduncle; mcp: middle cerebellar peduncle; sp5: spinal trigeminal tract; tz: trapezoid body.

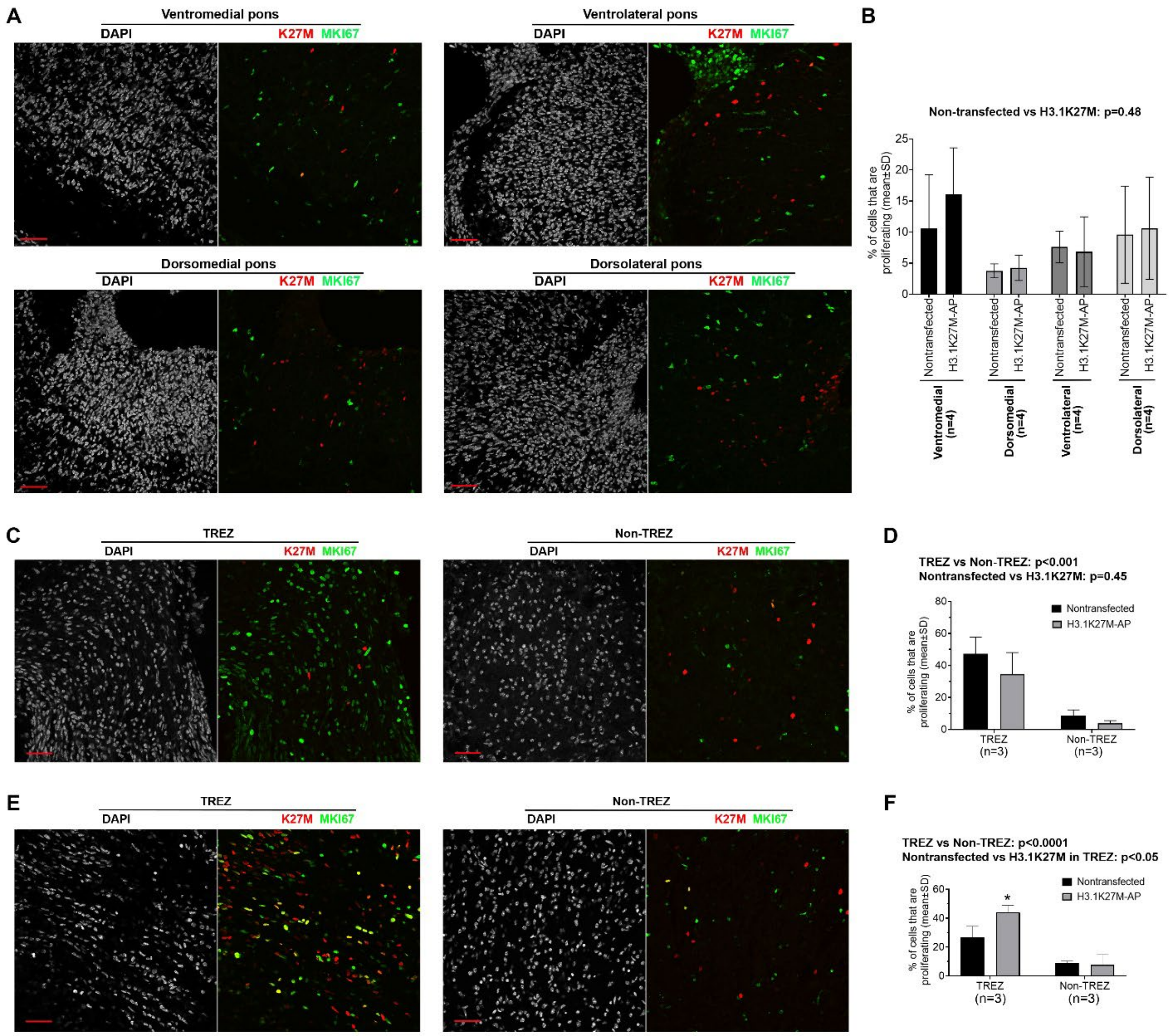

Figure S3. Proliferation and anatomical location of cells transfected with H3.1K27M-AP at different time points, related to Figures 3 and 5

Mouse embryos were electroporated with the H3.1K27M-AP plasmid mixture at E11.5. Proliferation of transfected cells (identified as K27M^{+} cells) at different anatomical locations was analyzed at the stages of E15.5 **(A and B)**, P4 **(C and D)**, and P11 **(E and F)**. At E15.5, the anatomical locations are divided into dorsomedial, dorsolateral, ventromedial, and ventrolateral pons for quantification because at this stage TREZ axon tracts are not fully formed.

Representative images are shown in (A), (C), and (E), and quantifications and statistics are shown in (B), (D), and (F), respectively. Cells transfected with H3.1K27M-AP (identified as $K27M^+$ cells) generally exhibited similar proliferation capacities to those of neighboring non-transfected cells (identified as $K27M^-$ cells), with the exception that transfected cells in the TREZ at P11 showed a moderate increase in the percentage of cells that were proliferating (F). Two-way ANOVA was used to analyze the effect of mutation (H3.1K27M-AP vs nearby non-transfected cells) and anatomical location on cell proliferation. No significant differences were found between H3.1K27M-AP and non-transfected cells at E15.5 (B) and at P4 (D). Scale bars: 20μm.

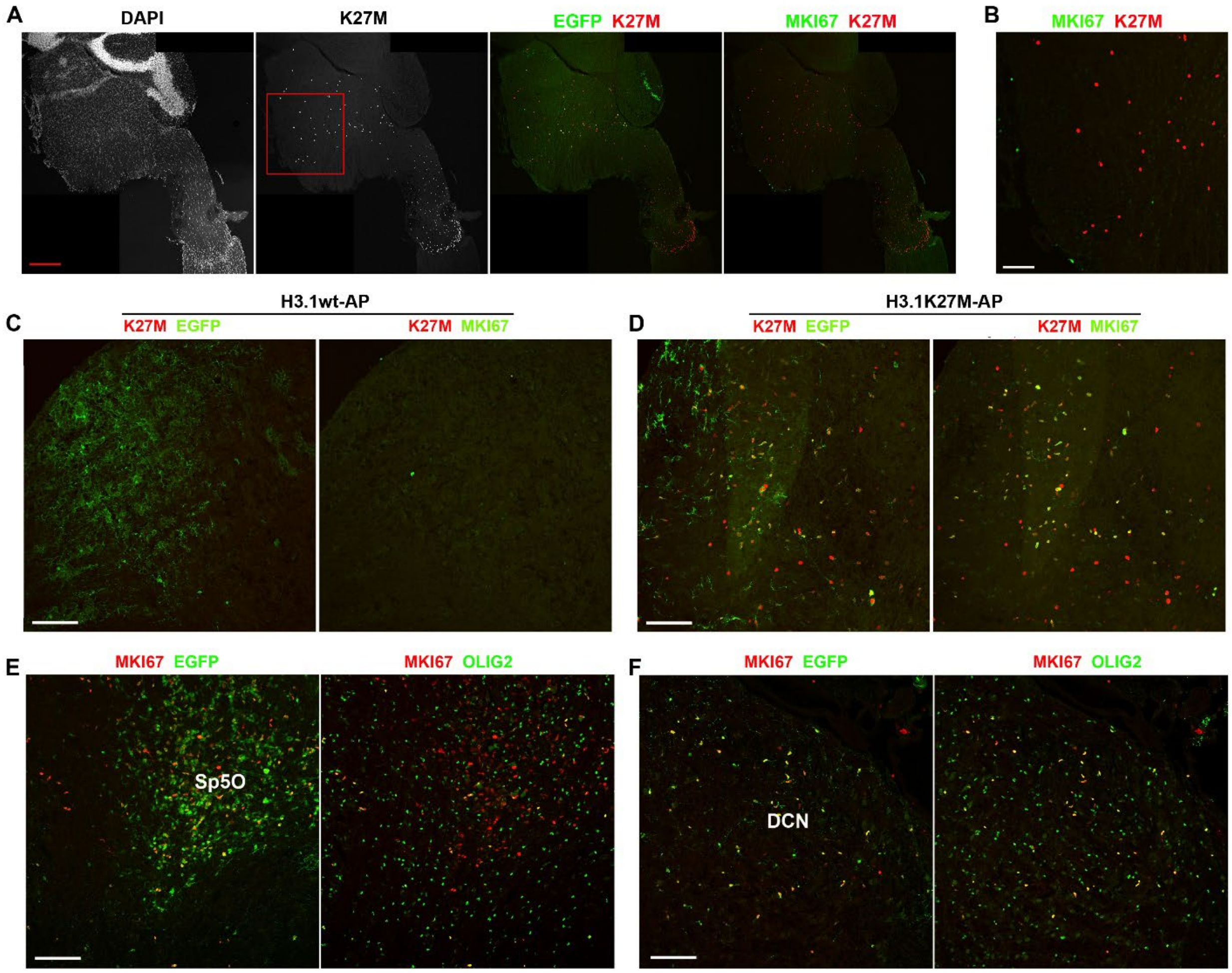

Figure S4. The effects of H3.3K27M-TPC expression on cell proliferation in the mouse brainstem, related to Figures 3 and 5

Transposon-based plasmids for expressing H3.3-K27M, TRP53-R172H, PDGFRA, and CCND2 (H3.3K27M-TPC) were introduced into the mouse brainstem via in utero electroporation at E11.5. In control experiments (the H3.3wt-TPC group), the plasmid for expressing H3.3-K27M was replaced with a plasmid for expressing wild-type H3.3.

(A and B) Expression of H3.3K27M-TPC at moderate levels does not induce cell proliferation in the mouse brainstem. Composite confocal images of the brainstem of a P52 mouse expressing H3.3K27M-TPC are shown in (A). EGFP was detected in few cells within the brainstem, and

K27M immunoreactivity revealed only a moderate number of positive cells within the brainstem. The boxed area in (A) is shown at higher magnification in (B). Note that most of the scattered proliferating cells in the brainstem are not transfected cells as these do not express K27M. Images are representative of seven mice at the age of P45-P183 transfected with H3.3K27M-TPC at moderate efficiencies. Scale bars: 300μm in (A), and 100μm in (B).

(C and D) High expression of H3.3K27M-TPC induces extensive cell proliferation in the brainstem. Confocal images show the cochlear nucleus areas from a P98 mouse of the control group and a P125 mouse of the H3.3K27M-TPC group. No K27M immunoreactivity and few proliferating cells were detected in the control section (C), whereas extensive proliferating K27M^{+} cells were detected in the H3.3K27M-TPC section (D). Images are representative of seven control mice with high transfection efficiencies at the age of P81-P165 and 10 mice of the H3.3K27M-TPC group with high transfection efficiencies at the age of P47-P163. Scale bars: 100μm.

(E and F) The clusters of proliferating cells expressing H3.3K27M-TPC include both OLIG2^{+} and OLIG2^{-} populations. Confocal images of proliferating cells in the brainstem of a P163 mouse expressing H3.3K27M-TPC are shown. An example of a cluster of proliferating cells that includes both OLIG2^{+} and OLIG2^{-} cells is shown in (E), and an example of a cluster of proliferating cells that are mostly OLIG2^{+} is shown in (F). Abbreviations are used as follows. DCN: dorsal cochlear nucleus; Sp5O: spinal trigeminal nucleus, oral part. Scale bars: 100μm.

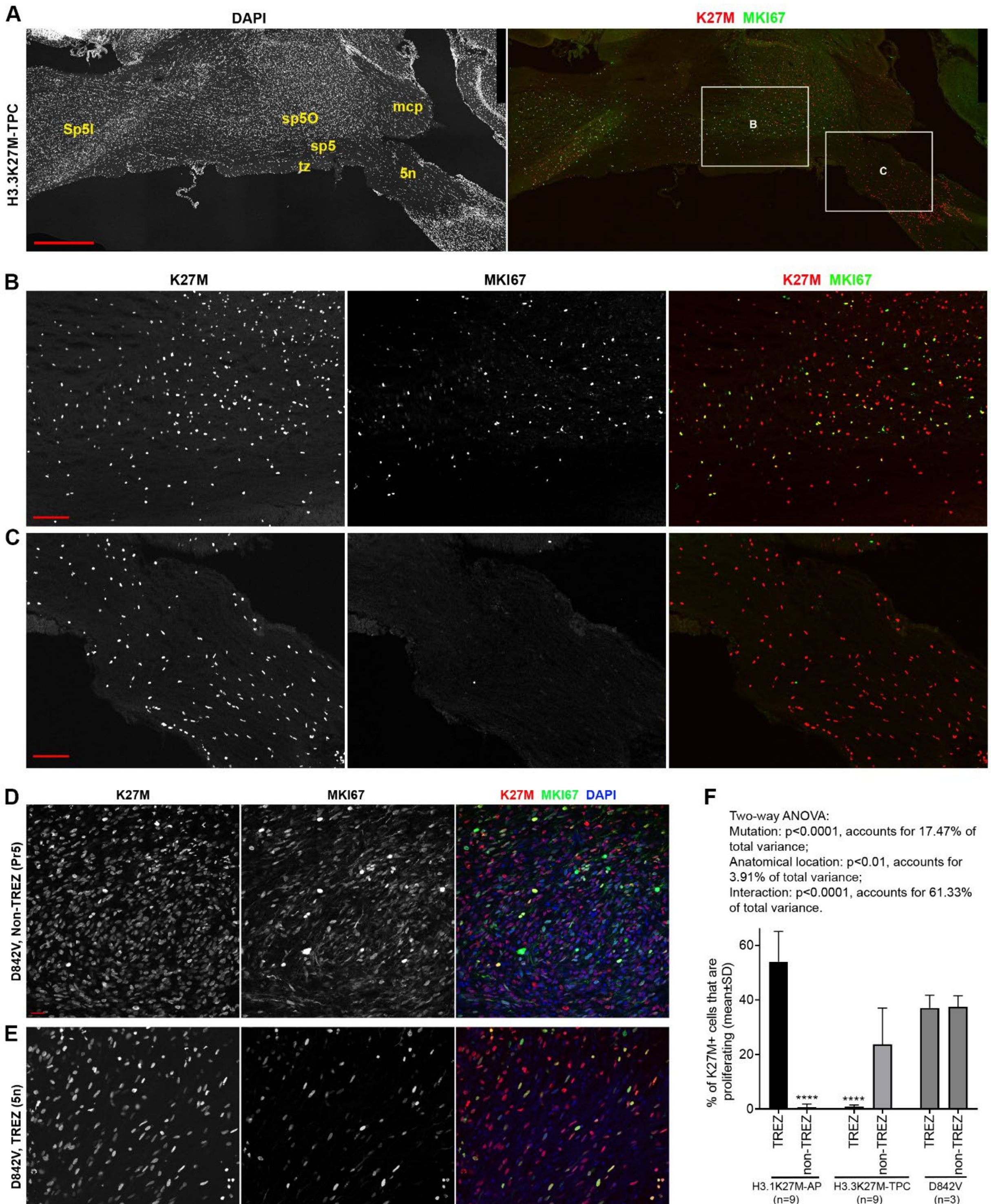

A
DAPI
K27M MKI67
H3.3K27M-TPC
Sp5I
sp5O
mcp
sp5
tz
5n
B
C
B
K27M
MKI67
K27M MKI67
C
D
K27M
MKI67
K27M MKI67 DAPI
D842V, Non-TREZ (Pr5)
E
D842V, TREZ (5n)
F
Two-way ANOVA:
Mutation: p<0.0001, accounts for 17.47% of total variance;
Anatomical location: p<0.01, accounts for 3.91% of total variance;
Interaction: p<0.0001, accounts for 61.33% of total variance.
% of K27M+ cells that are proliferating (mean±SD)
60
40
20
0
****
****
TREZ
non-TREZ
TREZ
non-TREZ
TREZ
non-TREZ
H3.1K27M-AP (n=9)
H3.3K27M-TPC (n=9)
D842V (n=3)

Figure S5. TREZ-specific glioma cell proliferation is genetic mutation-dependent, related to Figures 3 and 5

(A-C) Expression of H3.3K27M-TPC induces extensive cell proliferation outside of the TREZ but not in the TREZ. Composite confocal images of the brainstem of a P163 mouse expressing H3.3K27M-TPC are shown in (A). Proliferating cells are distributed extensively in both the pons and the medulla. Boxed areas in (A) are shown at higher magnifications in (B) (area outside of the TREZ) and (C) (area at the TREZ) to illustrate that extensive proliferation of $K27M^{+}$ cells occurs outside of the TREZ but not within the TREZ. Images are representative of 10 mice (P47-P163) transfected with H3.3K27M-TPC at high efficiencies. Scale bars: 500μm in (A) and 100μm in (B) and (C).

(D and E) Replacing the wild-type PDGFRA in the H3.3K27M-TPC mixture with an activated PDGFRA-D842V (the D842V group) results in rapid gliomagenesis throughout the brainstem, including both outside of the TREZ (D) and within the TREZ (E). Images are representative of three samples at the age of P21-P31. Scale bars: 10μm.

(F) Quantification of cell proliferation induced by different genetic mutations. Two-way analyses show a strong interaction between genetic mutation and anatomical location – thereby indicating that genetic context has a major effect on the anatomical specificity of cell proliferation. ****$p<0.0001$ (TREZ vs non-TREZ for H3.1K27M-AP and for H3.3K27M-TPC), two-way ANOVA.

Abbreviations -- 5n: trigeminal nerve; mcp: middle cerebellar peduncle; Pr5: principal sensory nucleus of the trigeminal nerve; sp5: spinal trigeminal tract; Sp5O: spinal trigeminal nucleus, oral part; Sp5I: spinal trigeminal nucleus, interpolar; tz: trapezoid body.

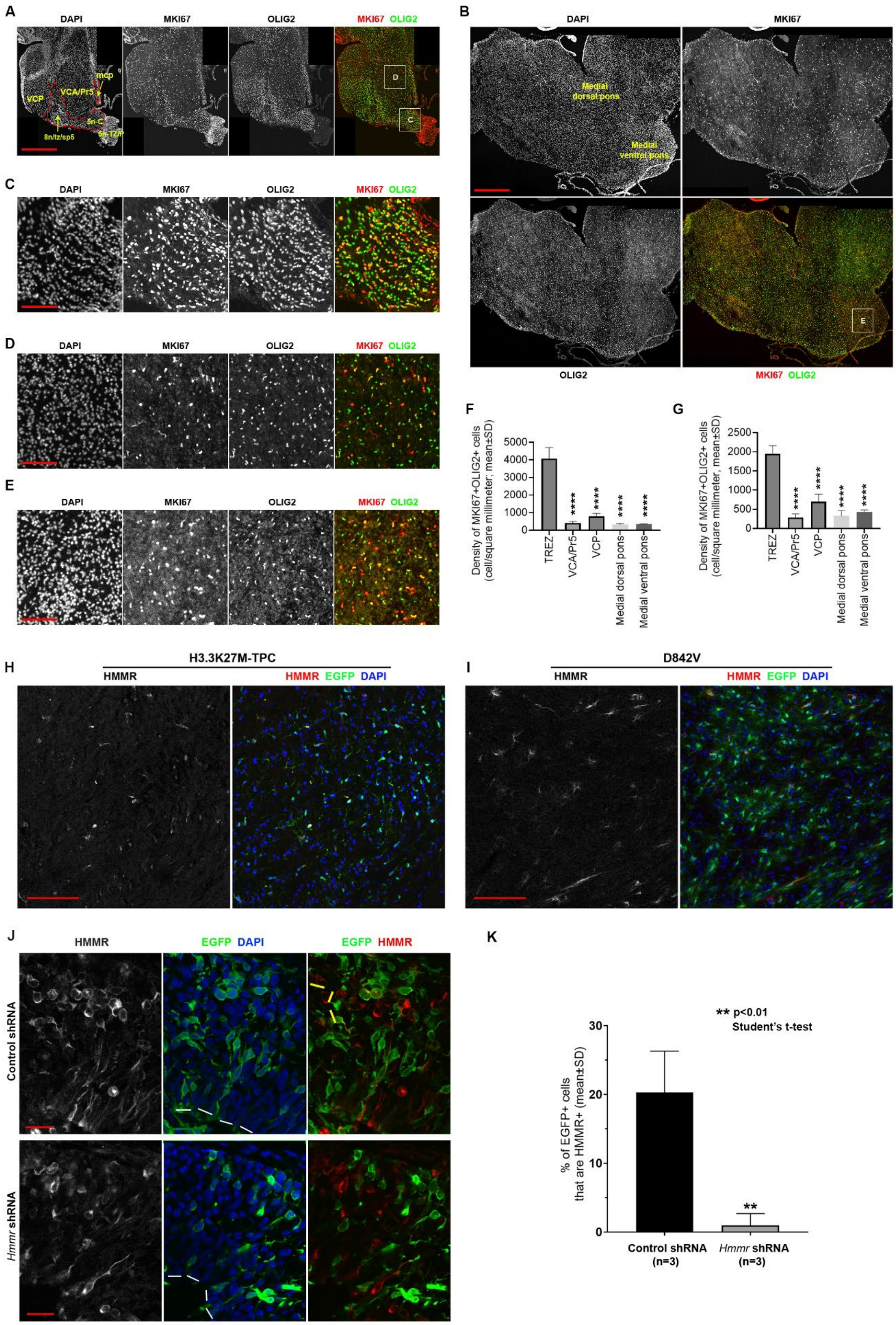
A
DAPI
MKI67
OLIG2
MKI67 OLIG2
VCP
VCA/Pr5
mcp
5n-C
5n-TZ/P
8n/tz/sp5
D
C
B
DAPI
MKI67
Medial dorsal pons
Medial ventral pons
E
OLIG2
MKI67 OLIG2
C
DAPI
MKI67
OLIG2
MKI67 OLIG2
D
DAPI
MKI67
OLIG2
MKI67 OLIG2
E
DAPI
MKI67
OLIG2
MKI67 OLIG2
F
Density of MKI67+OLIG2+ cells (cell/square millimeter, mean±SD)
5000
4000
3000
2000
1000
0
****
TREZ
VCA/Pr5
VCP
Medial dorsal pons
Medial ventral pons
G
Density of MKI67+OLIG2+ cells (cell/square millimeter, mean±SD)
2500
2000
1500
1000
500
0
****
TREZ
VCA/Pr5
VCP
Medial dorsal pons
Medial ventral pons
H
H3.3K27M-TPC
HMMR
HMMR EGFP DAPI
I
D842V
HMMR
HMMR EGFP DAPI
J
HMMR
EGFP DAPI
EGFP HMMR
Control shRNA
Hmmr shRNA
K
** p<0.01
Student's t-test
% of EGFP+ cells that are HMMR+ (mean±SD)
30
20
10
0
**
Control shRNA (n=3)
Hmmr shRNA (n=3)

Figure S6. Role of HMMR in TREZ gliomagenesis induced by H3.1K27M-AP, related to Figures 6 and 7

(A-G) The TREZ is characterized by a high density of proliferating oligodendrocyte-lineage cells at early postnatal stages. Representative composite images of the lateral mouse brainstem and the medial mouse brainstem at P4 are shown in (A) and (B), respectively. Boxed areas in (A) and (B) are shown at higher magnifications in (C; area 5n-c, part of TREZ), (D; area Pr5), and (E; medial ventral pons) to illustrate the high density of proliferating oligodendrocyte-lineage cells (i.e. $MKI67^{+}OLIG2^{+}$ cells) at the TREZ. Scale bars: 500μm in (A) and (B), 100μm in (C-E). Quantifications of the density of proliferating oligodendrocyte-lineage cells at different anatomical locations of the brainstem at P4 are shown in (F). Similar quantifications at the stage of P8 are shown in (G). For both (F) and (G), quantifications were performed on four embryos for each anatomical location (n=4). ****$p<0.0001$, one-way ANOVA.

(H and I) Expression of HMMR is detected in a subset of cells transfected with H3.3K27M-TPC, and in a subset of cells of the D842V group (i.e. the group transfected with the H3.3K27M-TPC mixture where the wild-type PDGFRA was replaced with the D842V mutant). Images are representative of three or more samples for each group. Scale bars: 100μm.

(J and K) Expression of an *Hmmr* shRNA construct reduces the numbers of transfected cells with HMMR immunoreactivity. A transposon-based control shRNA or *Hmmr* shRNA plasmid (together with an EGFP plasmid and a plasmid for expressing the Tol2 transposase) was introduced into the brainstem of mouse embryos at E11.5 via in utero electroporation. Electroporated embryos were sacrificed at E15.5, and HMMR expression in transfected cells at the pons was analyzed by immunostaining. (J) Representative confocal images. (K) Quantification. Compared to the control shRNA group, markedly reduced fractions of transfected cells ($EGFP^{+}$) in the *Hmmr* shRNA group showed HMMR immunoreactivity. **$p<0.01$ (Student's t-test, n values represent the number of embryos). Scale bars: 20μm.

Abbreviations -- 5n-c: central portion of the trigeminal nerve; 5n-TZ/P: transitional zone and peripheral portion of the trigeminal nerve; 6n: the 6th cranial nerve; 7n: the 7th cranial nerve; 8n: the 8th cranial nerve; mcp: middle cerebellar peduncle; PnO: pontine reticular nucleus, oral part; Pr5: principal sensory trigeminal nucleus; sp5: spinal trigeminal tract; tz: trapezoid body; VCA: ventral cochlear nucleus, anterior; VCP: ventral cochlear nucleus, posterior. The TREZ niche, outlined by dashed lines in (A), includes 5n-c and part of the mcp and 8n/tz/sp5 axon tracts that are close to 5n-c.

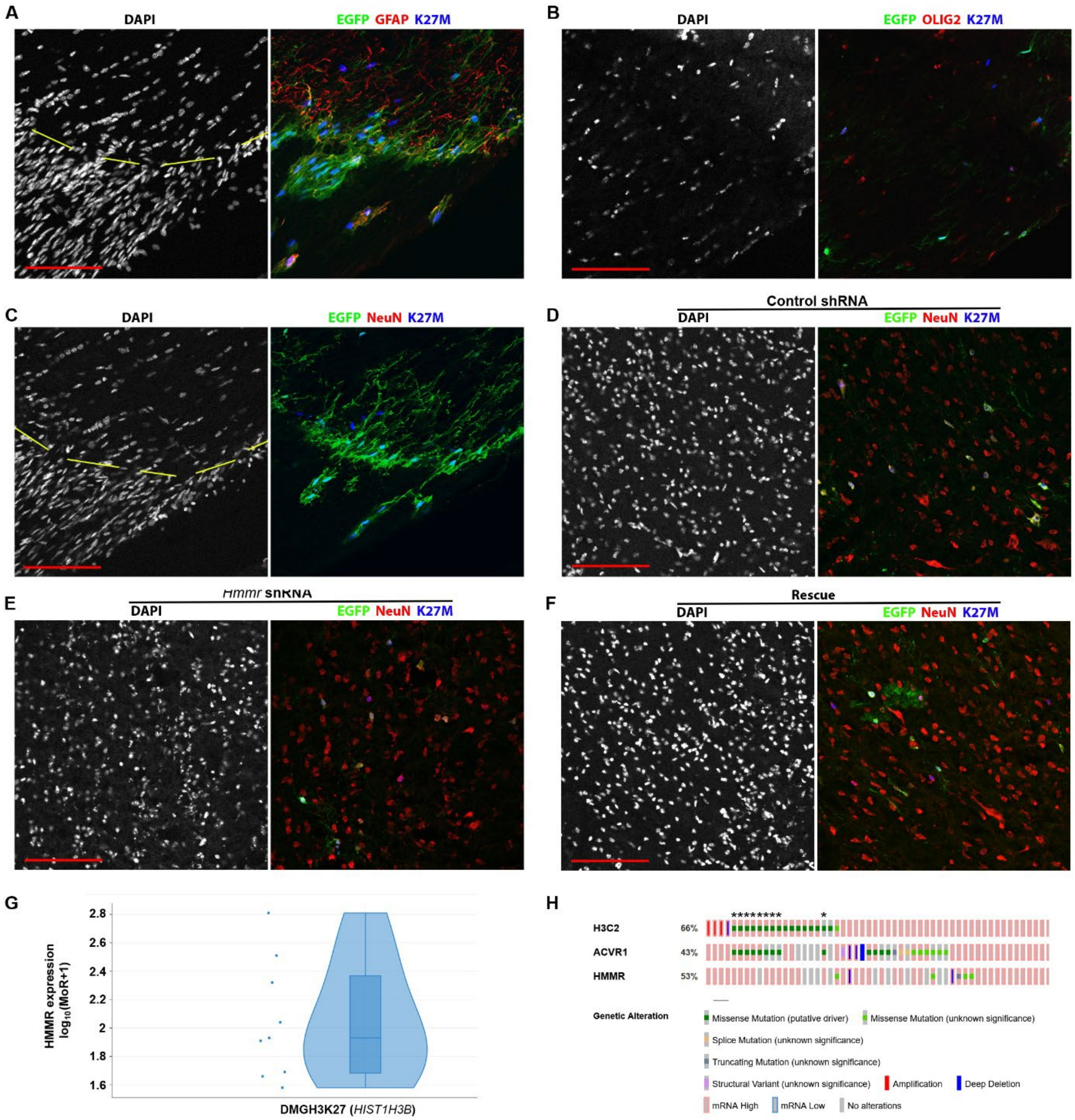

Figure S7. Role of HMMR in TREZ gliomagenesis induced by H3.1K27M-AP, related to Figures 6 and 7

(**A-C**) Cells on the trigeminal nerve transfected with H3.1K27M-AP (together with control shRNA, or *Hmmr* shRNA and a plasmid for HMMR expression) express glial cell markers. Confocal images from a control shRNA group (representative of three samples of the control

shRNA group and three samples of the rescue group) are shown. The identity of the transfected cells on the trigeminal nerve were determined by monitoring expression of GFAP, OLIG2, and NeuN. The border between the central domain of the trigeminal nerve and the peripheral domain of the trigeminal nerve is indicated by yellow dashed lines in (A) and (C). A subset of transfected cells expressed GFAP (A), and a subset of transfected cells expressed OLIG2 (B). No transfected cells expressed NeuN (C). Scale bars: 100μm.

(**D-F**) Nearly all K27M$^+$ cells in non-TREZ areas are neurons. In control (control shRNA + H3.1K27M-AP), *Hmmr* shRNA (*Hmmr* shRNA + H3.1K27M-AP), and rescue (*Hmmr* shRNA + H3.1K27M-AP + HMMR plasmid) groups, nearly all K27M$^+$ cells expressed the neuronal marker NeuN. Some EGFP$^+$ cells in both groups were NeuN$^-$. However, these cells were also K27M$^-$. Scale bars: 100μm.

(G and H) *HMMR* is expressed in human pediatric diffuse midline gliomas (DMGs) harboring the *H3.1K27M* mutation. (G) Search of the PeCan database through the St. Jude Cloud portal identified nine samples of DMG, H3K27-altered with the H3 mutation occurring in *HIST1H3B* (the alias used in this database for the *H3C2* gene that encodes the H3.1 protein). The Y-axis indicates the expression of *HMMR* mRNA in these samples. (H) Search of the PedcBioPortal database identified nine patients (indicated by asterisks) with co-occurring *H3.1K27M* (*H3C2* encodes the H3.1 protein) and an activating *ACVR1* mutation. Only part of the OncoPrint of the search result is shown in this panel. All of the nine patients with co-occurring *H3.1K27M* and *ACVR1* mutations were classified as DMG, H3K27-altered. Among these nine patients, eight displayed high *HMMR* mRNA levels relative to healthy tissues. The percentages indicate the fractions of patients with alterations in *H3C2*, *ACVR1*, or *HMMR*. Overall, 53% of patients (pediatric brain tumor patients; including 8 patients with co-occurring *H3.1K27M/ACVR1* mutations and 952 patients without co-occurring *H3.1K27M/ACVR1* patients) displayed high

HMMR mRNA levels (a small fraction of these samples also had other *HMMR* alterations in addition to high mRNA) relative to healthy tissues.

Table S1. Mice subjected to in utero electroporation for assessing gliomagenesis/glioma-like growth (P4 pups and embryos are not included), related to Figures 1-7 and S1-S7.

Group	Sex	Age	Location of IUE	Hydrocephalus (H) or small body size (S)	Head tilt (HT)	Glioma/glioma-like growth (G)
EGFP	M	P30	brainstem			
EGFP	F	P34	brainstem	H		
EGFP	M	P64	brainstem			
EGFP	M	P91	brainstem			
EGFP	M	P91	brainstem			
EGFP	F	P91	brainstem			
EGFP	F	P92	brainstem			
EGFP	F	P135	brainstem			
EGFP	M	P135	brainstem			
H3.1wt-AP	F	P60	brainstem			
H3.1wt-AP	F	P74	brainstem			
H3.1wt-AP	M	P74	brainstem	H		
H3.1wt-AP	M	P75	brainstem			
H3.1wt-AP	F	P90	brainstem			
H3.1wt-AP	M	P90	brainstem	H		
H3.1wt-AP	M	P159	brainstem			
H3.1wt-AP	M	P159	brainstem			
H3.1wt-AP	F	P159	brainstem			
H3.1wt-AP	F	P159	brainstem			
H3.1K27M-AP	M	P40	brainstem	H		G
H3.1K27M-AP	F	P42	brainstem		HT	G
H3.1K27M-AP	F	P43	brainstem		HT	G
H3.1K27M-AP	M	P45	brainstem	H		G
H3.1K27M-AP	M	P45	brainstem	H		G
H3.1K27M-AP	F	P49	brainstem		HT	G
H3.1K27M-AP	F	P52	brainstem		HT	G
H3.1K27M-AP	F	P53	brainstem		HT	G
H3.1K27M-AP	M	P56	brainstem		HT	G
H3.1K27M-AP	M	P57	brainstem	H		G
H3.1K27M-AP	F	P57	brainstem			G
H3.1K27M-AP	M	P60	brainstem	H		G
H3.1K27M-AP	F	P63	brainstem		HT	G
H3.1K27M-AP	M	P64	brainstem		HT	G
H3.1K27M-AP	M	P67	brainstem	H	HT	G
H3.1K27M-AP	M	P67	brainstem			G
H3.1K27M-AP	M	P90	brainstem			G
H3.1K27M-AP	M	P20	brainstem	H		G*
H3.1K27M-AP	M	P20	brainstem	H		G*
H3.1K27M-AP	F	P20	brainstem			G*
H3.1K27M-AP	F	P21	brainstem	H		G*
H3.1K27M-AP	F	P21	brainstem	H		G*
H3.1K27M-AP	M	P21	brainstem			G*
H3.1K27M-AP	M	P22	brainstem	H		G*
H3.1K27M-AP	M	P26	brainstem	H		G*
H3.1K27M-AP	F	P29	brainstem	H		G*
H3.1K27M-AP	M	P32	brainstem	H		G*
H3.1K27M-AP	M	P41	brainstem	H		G*
H3.1K27M-AP	F	P47	brainstem	H		G*
H3.1K27M-AP	M	P56	brainstem	H		G*
H3.1K27M-AP	F	P63	brainstem			G*

H3.1K27M-AP	M	P75	brainstem	H		G*
H3.1K27M-AP	F	P91	brainstem			G*
H3.1K27M-AP, HMMR	F	P28	brainstem	H		G
H3.1K27M-AP, HMMR	F	P60	brainstem			G
H3.1K27M-AP, HMMR	M	P61	brainstem	H	HT	G
H3.1K27M-AP, DN-HMMR	F	P21	brainstem	S		G**
H3.1K27M-AP, DN-HMMR	M	P22	brainstem	S		G**
H3.1K27M-AP, DN-HMMR	M	P22	brainstem	S		G**
H3.1K27M-AP, DN-HMMR	M	P53	brainstem	H	HT	G**
H3.1K27M-AP, DN-HMMR	M	P55	brainstem		HT	G**
H3.1K27M-AP, DN-HMMR	F	P60	brainstem	H		G**
H3.1K27M-AP, DN-HMMR	M	P60	brainstem			G**
H3.1wt-AP	F	P19	FB/MB	H		
H3.1wt-AP	F	P21	FB/MB	H		
H3.1wt-AP	F	P22	FB/MB	H		
H3.1wt-AP	M	P27	FB/MB	H		
H3.1wt-AP	F	P28	FB/MB	H		
H3.1wt-AP	F	P60	FB/MB			
H3.1wt-AP	M	P60	FB/MB			
H3.1wt-AP	F	P91	FB/MB			
H3.1wt-AP	F	P91	FB/MB			
H3.1K27M-AP	M	P21	FB/MB	H		
H3.1K27M-AP	M	P21	FB/MB			
H3.1K27M-AP	M	P23	FB/MB	H		
H3.1K27M-AP	F	P25	FB/MB			
H3.1K27M-AP	M	P25	FB/MB			
H3.1K27M-AP	M	P25	FB/MB			
H3.1K27M-AP	M	P25	FB/MB			
H3.1K27M-AP	M	P28	FB/MB	H		
H3.1K27M-AP	M	P29	FB/MB	H		
H3.1K27M-AP	M	P31	FB/MB	H		
H3.1K27M-AP	F	P47	FB/MB	H		
H3.1K27M-AP	M	P69	FB/MB	H		
H3.1K27M-AP	F	P73	FB/MB	H		G***
H3.1K27M-AP	M	P130	FB/MB	H		G***
H3.1K27M-AP	M	P135	FB/MB			
H3.1K27M-AP	F	P135	FB/MB			
H3.3wt-TPC	M	P81	brainstem	H		
H3.3wt-TPC	M	P98	brainstem	H		
H3.3wt-TPC	M	P98	brainstem			
H3.3wt-TPC	F	P98	brainstem			
H3.3wt-TPC	F	P98	brainstem			
H3.3wt-TPC	M	P165	brainstem			
H3.3wt-TPC	M	P165	brainstem			
H3.3K27M-TPC	F	P20	Brainstem			G*
H3.3K27M-TPC	F	P20	Brainstem			G*
H3.3K27M-TPC	M	P20	Brainstem	H		G*
H3.3K27M-TPC	F	P20	Brainstem			G*
H3.3K27M-TPC	M	P20	Brainstem			G*
H3.3K27M-TPC	M	P20	Brainstem			G*
H3.3K27M-TPC	F	P20	Brainstem			G*
H3.3K27M-TPC	M	P20	Brainstem			G*
H3.3K27M-TPC	M	P45	brainstem	H		
H3.3K27M-TPC	F	P47	brainstem	H		G*
H3.3K27M-TPC	F	P48	brainstem	H		G*
H3.3K27M-TPC	F	P48	brainstem			

H3.3K27M-TPC	F	P49	brainstem			G*
H3.3K27M-TPC	F	P52	brainstem			
H3.3K27M-TPC	F	P52	brainstem			G*
H3.3K27M-TPC	M	P52	brainstem	H		G*
H3.3K27M-TPC	M	P54	brainstem	H		
H3.3K27M-TPC	M	P97	brainstem	H		G*
H3.3K27M-TPC	M	P125	brainstem	H		G*
H3.3K27M-TPC	F	P163	brainstem			
H3.3K27M-TPC	F	P163	brainstem			G*
H3.3K27M-TPC	F	P163	brainstem			G*
H3.3K27M-TPC	M	P163	brainstem			
H3.3K27M-TPC	M	P163	brainstem			G*
H3.3K27M-TPC	F	P183	brainstem			
D842V	F	P21	brainstem	H		G
D842V	M	P29	brainstem		HT	G
D842V	M	P31	brainstem			G
H3.1K27M-AP	M	P11	brainstem			
H3.1K27M-AP	F	P11	brainstem			
H3.1K27M-AP	M	P11	brainstem			
H3.1K27M-AP-control shRNA	F	P49	brainstem			
H3.1K27M-AP-control shRNA	F	P61	brainstem	H		
H3.1K27M-AP-control shRNA	M	P84	brainstem			
H3.1K27M-AP-*Hmmr* shRNA	M	P47	brainstem			
H3.1K27M-AP-*Hmmr* shRNA	M	P55	brainstem	H		
H3.1K27M-AP-*Hmmr* shRNA	F	P61	brainstem			
H3.1K27M-AP-*Hmmr* shRNA	F	P61	brainstem	H		
H3.1K27M-AP-*Hmmr* shRNA	F	P90	brainstem			
H3.1K27M-AP-*Hmmr* shRNA	F	P90	brainstem			
H3.1K27M-AP-*Hmmr* shRNA	M	P90	brainstem			
H3.1K27M-AP-*Hmmr* shRNA	F	P91	brainstem			
H3.1K27M-AP-*Hmmr* shRNA	F	P91	brainstem			
H3.1K27M-AP-*Hmmr* shRNA	M	P91	brainstem			
H3.1K27M-AP-*Hmmr* rescue	F	P46	brainstem			
H3.1K27M-AP-*Hmmr* rescue	M	P46	brainstem			
H3.1K27M-AP-*Hmmr* rescue	M	P53	brainstem			

G: Extensive glioma cell proliferation with apparent tumor bulk
G*: Extensive glioma/glioma-like cell proliferation without apparent tumor bulk.
G**: Proliferating tumor cells were those with low expression of DN-HMMR.
G***: Ventricular/periventricular gliomagenesis.
FB/MB: Forebrain/midbrain
